# Biofilm communities above and below the cuff of endotracheal tubes are spatially homogenous

**DOI:** 10.1186/s12931-025-03485-2

**Published:** 2026-01-22

**Authors:** Gisli G. Einarsson, Sujata Das, Jonathan A. Silversides, Nerielle Fundano, Elliott Lonsdale, Ronan McMullan, Daniel F. McAuley, Nicola J. Irwin, Colin P. McCoy, Matthew P. Wylie, Laura J. Sherrard

**Affiliations:** 1 School of Pharmacy, Queen’s University Belfast, Belfast, UK; 2 Wellcome-Wolfson Institute for Experimental Medicine, Queen’s University Belfast, Belfast, UK; 3 Department of Critical Care, Belfast Health and Social Care Trust, Belfast, UK

**Keywords:** Endotracheal tubes, Intensive care units, Biofilms, Microbiome

## Abstract

**Background:**

During mechanical ventilation, the inflated cuff of endotracheal tubes (ETTs) may help to minimise patient secretions containing microorganisms from reaching the lower trachea and distal ETT surface. The primary study aim was to determine if there were differences in biofilm formation above and below the cuff using microbial community profiling.

**Methods:**

In this prospective observational study, ETTs were collected following extubation of mechanically ventilated critically ill adults. The biofilm community composition and structure on ETT segments were examined by culture-independent (Illumina MiSeq 16S rRNA marker-gene sequencing) and -dependent (extended-quantitative culture) methods and related to clinical variables.

**Results:**

Sixty-four participants were recruited following a median (IQR) of 3.77 (1.56–7.49) days intubation. Although participant-level differences in communities were observed, most had evidence of multispecies biofilm development on the ETT surface. Community profiling indicated that there was no difference in alpha- and beta-diversity metrics or density in paired segments from above and below the ETT cuff. Culture-independent analysis detected a greater biofilm richness and more often identified microorganisms reported with clinically-directed culture of respiratory secretions than extended-quantitative culture (82.35% vs. 52.94%). At extubation a higher community density was associated with increased richness (*p* = 0.01) and diversity (*p* = 0.03) and reduced evenness (*p* = 0.01) and dominance (*p* = 0.03), but not bacterial pathogen or yeast presence/abundance by culture. Participant characteristics were not associated with the extent of biofilm community density.

**Conclusions:**

Biofilm communities forming above and below the ETT cuff are highly individual but conserved suggesting that an inflated ETT cuff does not provide an effective microbial barrier.

**Supplementary Information:**

The online version contains supplementary material available at 10.1186/s12931-025-03485-2.

## Introduction

Mechanical ventilation is used in intensive care units (ICUs), most commonly for critically ill patients in acute respiratory failure and is typically facilitated by tracheal intubation with a cuffed endotracheal tube (ETT). A potential complication of mechanical ventilation is the development of ventilator-associated pneumonia (VAP), which may occur in up to ~25% of patients and has an attributable mortality rate of ~10% [1].

In endotracheally-intubated patients the cough reflex is suppressed providing the opportunity for microorganisms from the oropharynx, subglottic area, sinuses and gastrointestinal tract to gain access to the lungs via repeated microaspiration [2]. Although an adequately inflated ETT cuff, which sits below the larynx, creates a seal against the tracheal wall, pooled secretions containing microorganisms may still pass around the cuff [2].

Furthermore, microorganisms may colonise the outer and inner luminal surface of ETTs within hours of mechanical ventilation and form a biofilm [3]. The ETT biofilm is a potential source of infection that could lead to the development of or relapse of VAP through, for example, detachment and migration of biofilm aggregates into the lungs [4]. Studies have shown that the same pathogen is often detected in oropharyngeal secretions or tracheal aspirates and ETT biofilms [5–8]. Culture-independent analyses have also revealed a greater diversity of microorganisms in ETT biofilms, including commensals, than is targeted by standard culture-dependent techniques but the role of these polymicrobial communities in VAP is unclear [3, 9–15]. An improved understanding of the composition and structure of the microbial communities may help to inform new strategies to prevent biofilm formation on the ETT surface.

Given that the ETT cuff is deemed critical for reducing secretions containing microorganisms from reaching the lower airways and distal ETT surface, the primary aim of this study was to assess if the biofilm ecology differed above and below the cuff, in a prospective observational study of an ICU patient cohort utilising microbial community profiling. We also sought to determine if there were possible associations between patient characteristics and the ETT biofilm community at extubation.

## Methods

Additional details are provided in the Supplementary Materials.

### Participants

Adult patients (≥18 years) undergoing invasive mechanical ventilation with ETTs in the ICUs at the Royal Victoria Hospital and Belfast City Hospital within the Belfast Health and Social Care Trust, Northern Ireland (UK), were recruited between April 2023 and March 2024. These are general ICUs providing care for a broad range of critically ill patients other than solid organ transplant or cardiac surgical patients. An exclusion criterion was known or suspected infection with hazard group 3 or 4 biological agents. Standard measures for VAP prevention e.g. elevation of the head of the bed, tracheal suctioning, and ETT cuff pressure checks were performed routinely in keeping with clinical guidelines.

### ETT collection and processing

**Fig. 1:**
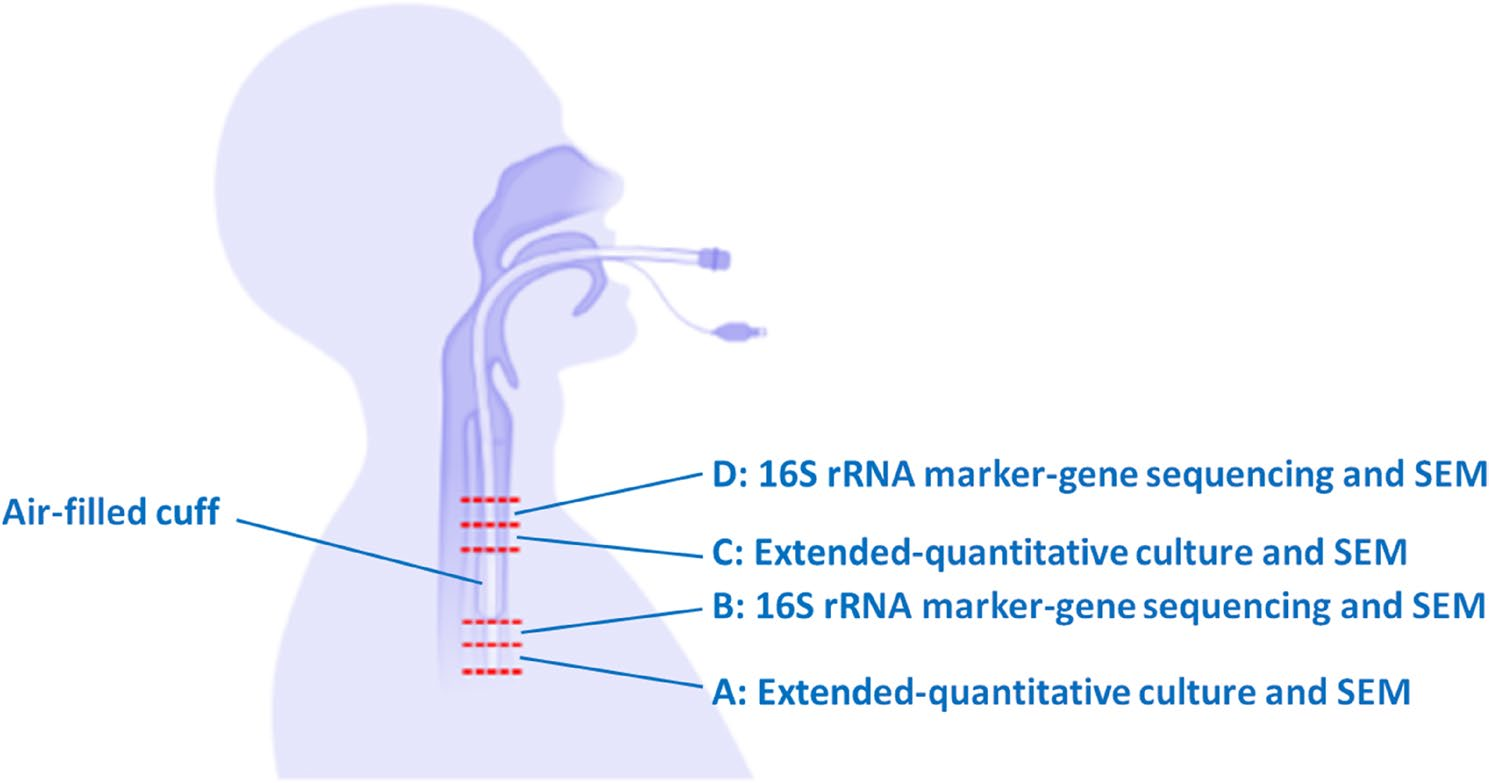
Schematic overview of endotracheal tube sectioning. Four cross-sectional sections (1-cm lengths labelled A, B, C and D) from the subglottic region above or the distal region below the cuff margin were excised. Sections were divided longitudinally into two pieces for subsequent biofilm assessments (eight segments in total) which included scanning electron microscopy (SEM), extended-quantitative culture and Illumina MiSeq 16S rRNA marker-gene sequencing. Figure created using BioRender.com

Following extubation, ETTs were immediately placed into a sterile specimen bag with an anaerobic air-generating paper sachet (AnaeroGen™) and sealed. Participant ETTs were processed as soon as possible following extubation, aiming for 24-hours or less. Four sections (1-cm cross-sectional lengths) from the subglottic region above or the distal region below the cuff margin were excised (Fig. 1 and Figure S1A). Sections were then divided longitudinally, using a sterile scalpel, into two pieces for subsequent analyses (eight segments in total). Four segments (two above and two below the cuff) were placed in 3% glutaraldehyde diluted with phosphate buffered saline (PBS) and incubated at 4°C overnight to fixate the biofilm to the ETT surface for subsequent imaging. Biofilm microorganisms were dislodged from the ETT surface of the remaining segments (two above and two below the cuff) in 10 mL quarter-strength Ringers Solution (QSRS) using a vortexing-sonication-vortexing method. The sonicate was concentrated by centrifugation at 3000 g and re-suspended in 1 mL QSRS for Illumina MiSeq 16S rRNA marker-gene sequencing or extended-quantitative culture.

### Scanning electron microscopy

Segments fixed with the glutaraldehyde solution were washed and then progressively dehydrated in a graded series of ethanol dilutions. Samples were dried, mounted on aluminum stubs and sputter coated with a thin Au film (~15–20 nm) [16]. Surface biofilm development was assessed using a Hitachi High Technologies TM3030 (Berkshire, UK) scanning electron microscopy (SEM) with an accelerating voltage of 15 kV. For each ETT, segments (above and below the cuff) were examined, and images representative of the typical field of view selected.

### Illumina MiSeq 16S rRNA marker-gene sequencing

Illumina MiSeq 16S rRNA marker-gene sequencing of ≥2 segment suspensions above and below the cuff was performed according to a standard Illumina workflow, except for using custom sequencing primers from the Earth Microbiome Protocol targeting the V4 hypervariable region [17]. Briefly, the paired raw.fastq files were imported in R (v4.1.2) and processed using the DADA2 pipeline (v1.22), followed by taxonomy assignment of amplicon sequence variants (ASVs) using the SILVA v138 reference database with species-level resolution [18]. Raw sequencing data was analysed for potential reagent contaminants using the decontam package (v1.14.0) in R (v4.1.2) followed by removal of the potential contaminant ASVs [19]. The quality controlled MiSeq sequencing data was next converted into a phyloseq-object and rarefied to 10,000 amplicons per sample prior to downstream analysis.

### Extended-quantitative culture

Extended-quantitative culture of two segment suspensions (one above and one below the cuff) was performed with all distinct colony morphologies enumerated and pure cultures identified to the species-level, if possible. The estimated density (total viable count [TVC]) of the biofilm community of each segment was based on the combined count of unique taxa identified and expressed as colony forming units per cm of tube segment length (CFU/cm).

### Antimicrobial susceptibility

A selection of bacterial species (59 non-duplicate participant isolates) underwent antimicrobial susceptibility testing using the disk diffusion method with results interpreted according to standard guidelines [20]. The panel of antibiotics used reflected those that were administered to participants during the study period.

### Participant characteristics

Age, sex, primary diagnosis on ICU admission, comorbidities, duration of mechanical ventilation, antibiotic use and diagnosis of VAP during mechanical ventilation were documented. A Charlson Comorbidity Index score was calculated [21]. Data on clinically-directed culture of respiratory secretions (tracheal aspirate; bronchoalveolar lavage; pleural fluid) were collected, if available.

### Ecological measurements

From the culture-independent and -dependent datasets, ecological measurements were completed. Alpha-diversity (both datasets) was assessed using four metrics: taxonomic richness, the total number of observed species; Shannon-Wiener index, accounting for both richness and evenness; Pielou’s evenness, measuring the uniformity of species abundances; and dominance, indicating the relative abundance (RA) of the most prevalent species. The RA of taxa within each ETT segment was determined based on either ASV counts (Illumina MiSeq dataset) or TVCs (extended-quantitative culture dataset). Beta-diversity (Illumina MiSeq dataset) was assessed using Bray-Curtis dissimilarity on Hellinger-transformed count data.

### Data analyses

A Pearson Chi-Square test with Yate’s Continuity correction applied or Fisher’s exact Test was used to compare dichotomous variables while a Mann-Whitney U, Wilcoxon-signed rank or Friedman test was used to compare continuous variables between groups.

Given that the density of microorganisms is used as an endpoint when assessing clinical samples microbiologically, extended-quantitative culture features of the biofilm and characteristics of participants were compared between those with an estimated ETT biofilm community density of >10^3^ CFU/cm or ≤10^3^ CFU/cm on the segment above the cuff. A TVC cut-off value of 10^3^ was chosen to reflect the geometric mean biofilm community density of tube segments; it was also a primary endpoint used in a previous study of tracheal aspirates [22].

Analyses were performed in R (v4.1.2), RStudio (v2021.09.2) and IBM SPSS Statistics (v30.0) with a p-value < 0.05 considered statistically significant.

## Results

### Cohort

Participant characteristics (*n* = 64) are summarised in Table 1 and are representative of a general cohort of critically ill adults. The median (IQR) duration of tracheal intubation was 3.77 (1.56–7.49) days with most participants (*n* = 55; 85.94%) receiving ≥1 antibiotic during this period. For 55/64 (85.94%) participants, ETTs were processed within 24-hours.

**Table 1:** Characteristics of study participants

Variable	Cohort
	Cohort (*n* = 64)
**ICU Site; n (%)**	
Royal Victoria Hospital	27 (42.19)
Belfast City Hospital	37 (57.81)
**Participant characteristic**	
Age, years; median (IQR)	61.05 (49.25–72.00)
Sex, male; n (%)	43 (67.19)
Comorbidity:	
• Any documented, n (%)	58 (90.63)
• Respiratory; n (%)	10 (15.63)
• Gastrointestinal; n (%)	11 (17.19)
• CCI score; median (IQR)	2.00 (1.00–4.00)
Primary diagnosis on ICU admission*; n (%)	
Cardiovascular	6 (9.38)
Gastrointestinal	18 (28.13)
Neurological	20 (31.25)
Respiratory	13 (20.31)
Other	7 (10.94)
**Clinical diagnosis of pneumonia on ICU admission; n (%)**	20 (31.25)
**Tracheal intubation**	
Duration; n (%):	
• <24 h	9 (14.06)
• 1–5 days	29 (45.31)
• >5 days	26 (40.63)
Duration, days; median (IQR)	3.77 (1.56–7.49)
Antibiotic administration:	
• ≥1 antibiotic; n (%)	55 (85.94)
• Total number; median (IQR)	2.00 (1.00–3.00)
Diagnosis of VAP; n (%)	7 (10.94)

Definitions: *ICU* intensive care unit, *CCI* Charlson Comorbidity Index, *VAP* ventilator-associated pneumonia

*Table S1 details each individual primary diagnosis on ICU admission

A summary of the number of ETTs included in the various biofilm assessments is shown in Figure S1B.

### ETT biofilm formation

**Fig. 2:**
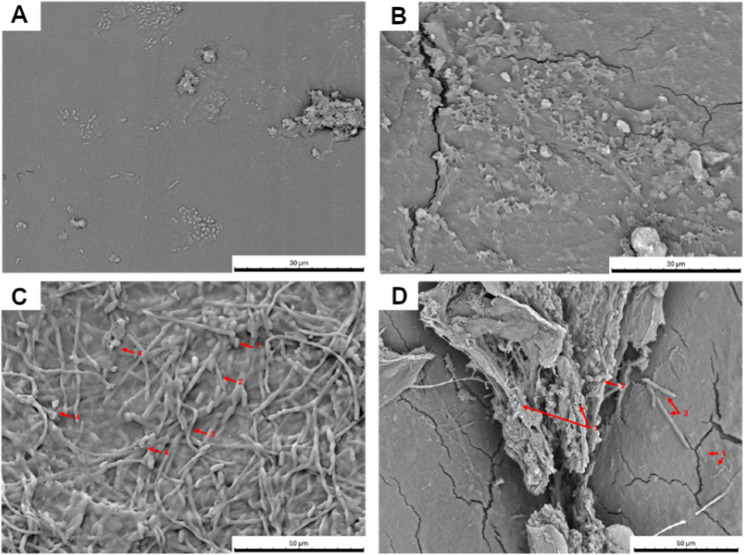
Representative scanning electron micrographs of endotracheal tube segments showing a mixture of morphological forms of biofilms developed. **(A)** Individual bacterial cells forming isolated clusters (BCH025). **(B)** Dense bacterial clusters (RVH022). **(C)** Presence of yeast cells and hyphae (BCH024). Arrows indicate: 1 = yeast cell; 2 = pseudohyphae; 3 = hyphae; 4 = bacterial cells. **(D)** Biofilm containing a mixture of bacterial and fungal cells (RVH013). Arrows indicate: 1 = bacterial cells; 2 = yeast cells; 3 = pseudohyphae and hyphae.

Evidence of biofilm formation was observed on the outer surface of 31/32 (96.88%) participant ETTs examined including segments above and below the cuff. Generally, the appearance of the biofilm matrix was variable from disperse clusters of bacteria (Fig. 2A) to dense, multispecies populations (Fig. 2B). Colonies and individual microbial cells were observed within the biofilm, including the presence of yeast cells and hyphae either in isolation (Fig. 2C) or forming cross-kingdom biofilms (Fig. 2D).

### Culture-independent analysis of biofilm communities

**Fig. 3:**
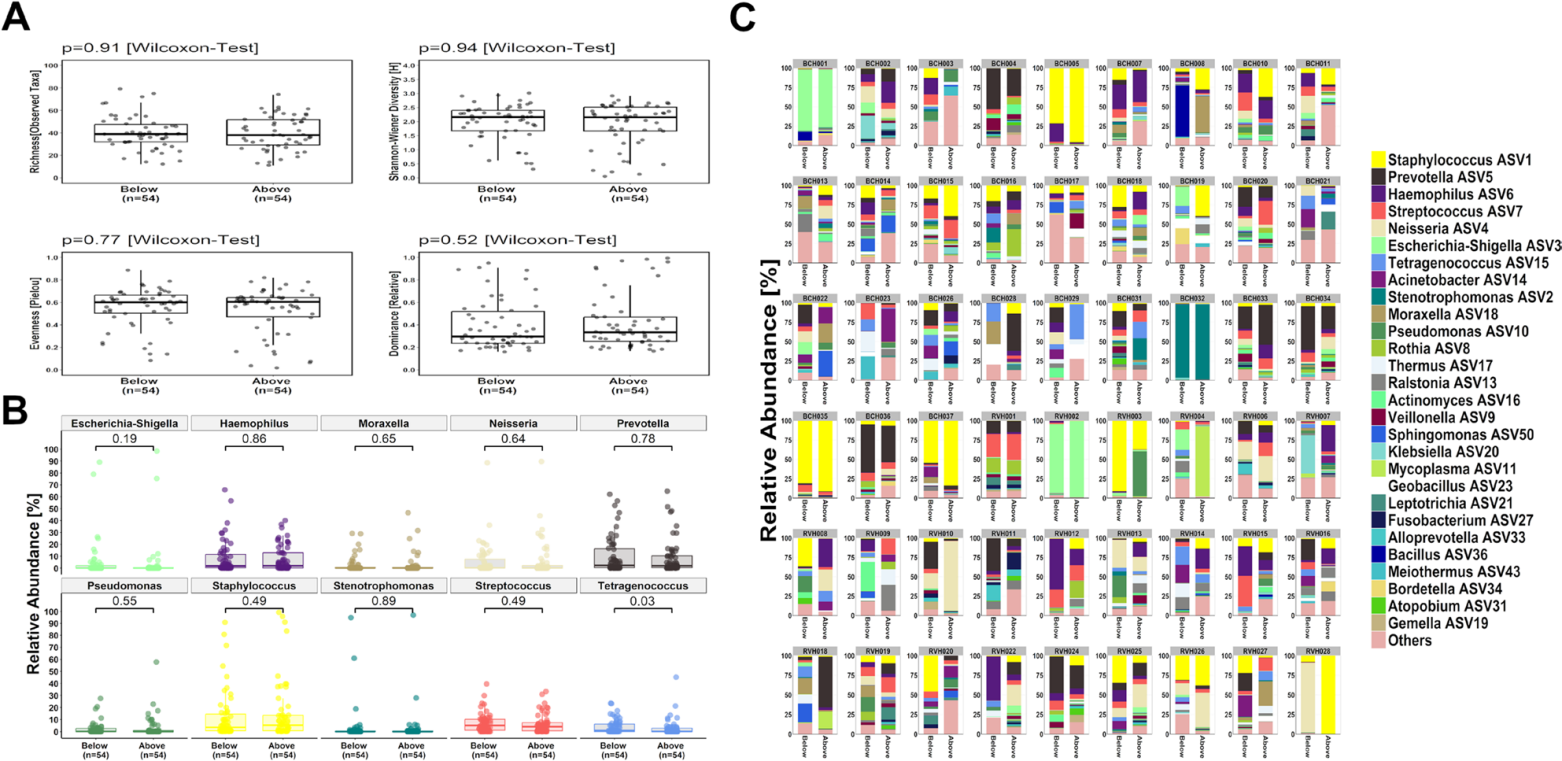
Comparison of within-participant (*n* = 54) endotracheal tube biofilm community characteristics in segments above and below the cuff. **(A)** Alpha-diversity metrics: taxonomic richness [S], community diversity (Shannon Wiener Index [H]), evenness [Pielou] and dominance [Relative]. **(B)** The relative abundance (%) of the top 10 genera detected. **(C)** Relative abundance (%) of genera for individual participants. In the box-and-whisker plots, the line inside the box indicates the median and the top and bottom of the box indicates the 25th and 75th percentile, respectively. The whiskers indicate the 90% confidence interval.

Most participants (*n* = 54/64; 84.38%) had paired ETT segments available for MiSeq 16S rRNA marker-gene comparison (inclusion threshold: 10,000 reads). There was no statistical difference (*p* > 0.05) in the alpha-diversity metrics assessed between segments above and below the cuff (median [IQR], above vs. below cuff: taxonomic richness, 39.00 [29.30–51.80] vs. 38.00 [32.00–47.50 ] ; Shannon-Wiener Index, 2.16 [1.67–2.52] vs. 2.17 [1.67–2.40]; evenness [Pielou], 0.60 [0.47–0.64] vs. 0.60 [0.50–0.66]; dominance, 0.33 [0.25–0.47] vs. 0.30 [0.24–0.52]; Fig. 3A). The most common genera identified included *Staphylococcus* spp., *Prevotella* spp. and *Haemophilus* spp. (Fig. 3B**).** Of the top 10 genera identified, only *Tetragenococcus* spp. had a significantly higher RA found below the cuff (*p* = 0.03, Fig.^3^B). Although inter-participant variation was observed with differences in the taxa identified and their RA (Fig. 3C), the within-ETT biofilm communities clustered closely for most individuals; however, community disparity between segments (i.e. communities within different clusters) was apparent for others (Figure S2). Nevertheless, as shown on the principal coordinate analysis plot there was a lack of separation of the communities above and below the cuff with no significant difference found between paired segments (Figure S3 [beta-diversity]; R² = 0.01, F = 0.48, *p* = 0.98). Furthermore, there were 7/9 ETTs which were stored for 1–5 days prior to processing and met the inclusion read threshold for subsequent Illumina MiSeq analysis; their biofilm community composition was representative of the ETTs sampled (Figure S2).

In addition, there were 18 participants who had four segments/ETT (two above and below the cuff) that underwent successful Illumina MiSeq analysis. The results corroborated with prior findings - there was limited variation in the matched ETT biofilm community compositions (Figure S4). Among those who were diagnosed with VAP during intubation and had ETT sequencing results available (*n* = 6), no difference (*p* > 0.05) in the biofilm community composition at extubation was found within paired ETT segments (Figure S5A). When compared to ETTs from participants without a VAP diagnosis (*n* = 48), a modest difference in dominance of the microbial community was observed above the cuff (median [IQR], VAP vs. VAP-Free: 0.22 [0.19–0.22] vs. 0.35 [0.27–0.47]; *p* = 0.03); however, there were no differences (*p* > 0.05) for the other alpha-diversity metrics measured (Figure S5B). Likewise, the ETT microbial communities below the cuff were similar regardless of VAP diagnosis status (Figure S5C).

Next, we explored variables that may contribute to the biofilm community structure (Figure S6). There was minimal variation between biofilm communities when stratified by ICU site. A higher richness, Shannon-Wiener Index and evenness [Pielou] were more likely when taxa such as *Haemophilus* spp., *Rothia* spp., *Streptococcus* spp. or *Prevotella* spp. were present. Conversely, dominance was associated with communities harbouring *Pseudomonas* spp. or *Staphylococcus* spp.; longer intubation duration and an increased number of antibiotics administered were also associated with dominance of these genera. However, the strength of the correlations between the variables was weak (ρ < 0.3; adjusted *p* > 0.05).

### Viable biofilm communities

Following extubation, 40/64 (62.50%) participant ETT segments were cultured (two segments/ETT) under aerobic and anaerobic conditions. Bacteria and/or fungi were cultured from 73/80 (91.25%) ETT biofilm communities. No growth was observed with both ETT segments from two participants and with one segment (below the cuff) from three participants. Of 455 isolates preserved, 92 different taxa were identified with four found in >10 biofilms (*Candida albicans*, *n* = 18; *Staphylococcus epidermidis*, *n* = 16; *Nakaseomyces glabrata*, *n* = 12; *Staphylococcus aureus*, *n* = 11; Table S2). There was no difference in the community density between segments (geometric mean [95% CI], above vs. below cuff: 1.51 × 10^3^ [4.41 × 10^2^ – 5.17 × 10^3^] CFU/cm vs. 1.41 × 10^3^ [3.65 × 10^2^ – 5.45 × 10^3^] CFU/cm; *p* = 0.82, Figure S7).

There was a median (IQR) biofilm taxonomic richness of 2.00 (1.00–6.00) in communities above and 2.00 (1.00–4.75) below the cuff (*p* = 0.27, Figure S8A) with no difference in the community diversity between segments (Shannon-Wiener index, median [IQR], above vs. below cuff: 0.63 [0.00–1.21] vs. 0.63 [0.03–1.01]; *p* = 0.93, Figure S8A). Likewise, community evenness (Pielou, median [IQR], above vs. below cuff: 0.82 [0.47–1.00] vs. 0.69 [0.43–0.94]; *p* = 0.20, Figure S8A) and dominance (median [IQR], above vs. below cuff: 0.59 [0.41–1.00] vs. 0.65 [0.43–0.99]; *p* = 0.72, Figure S8A) were analogous. It was also observed that the within-participant ETT biofilm community compositions were visually similar for most individuals based on the RA of taxa (Figure S8B).

Fungal taxa (*Candida* spp., *Nakaseomyces glabrata*, *Pichia kudriavzevii*; Table S2) were identified in 19/40 (47.50%) communities above and 14/40 (35.00%) below the cuff. When present, their RA was usually >1% (median [IQR], above vs. below cuff: 19.38% [3.35–61.76%] vs. 29.82% [1.81–99.46%]; *p* = 0.90). Using an anaerobic atmosphere, obligate anaerobes, e.g. *Prevotella* spp., were also recovered from biofilm communities of 14 participants’ ETT [above vs. below cuff: *n* = 12/40 (30.00%); *n* = 10/40 (25.00%)] with a range of 1–4 anaerobic species/segment.

### Clinically-directed culture

A comparison of clinically-directed culture of respiratory secretions to Illumina MiSeq 16S rRNA marker-gene sequencing and extended-quantitative culture results is provided in **Table S3**. Some bacteria were not speciated by the microbial community profiling methods used.

Results of clinically-directed culture and ETT biofilm culture were available for 20 participants – pathogens were reported in 17 respiratory secretions, 9 (52.94%) of whom potentially had the same pathogen cultured from their ETT with a median (IQR) RA of 70.35% (32.49–98.04%). The median (range) timing of respiratory secretion collection was −3 days (−9 to +5 days) relative to extubation in these nine participants. For those participants (*n* = 11) where culture results differed, the median (range) timing between collection of samples was similar (respiratory secretion collected −3 [−7 to +10] days relative to extubation; *p* = 0.70).

Among the 17 participants with pathogens reported, Illumina MiSeq 16S rRNA marker-gene sequencing provided evidence of taxa that potentially matched the clinically-directed culture in 14 (82.35%) participants, albeit in a low RA (<1%) in some cases. Of the seven participants diagnosed with VAP, five had clinically-directed culture results available. The same pathogen was found more often by Illumina MiSeq 16S rRNA marker-gene community profiling compared to extended-quantitative culture (80.00% vs. 20.00%). Furthermore, a pathogen (*S. aureus*; *Haemophilus influenzae*; *C. albicans*) was detected in three participants’ ETT communities when ‘no growth’ was reported by clinically-directed culture.

Finally, susceptibility results from clinically-directed culture of respiratory secretions showed that a higher proportion of respiratory isolates tested were susceptible to all antibiotics tested (*n* = 8/25, 32.00%) compared to ETT isolates (*n* = 9/59, 15.25%); however, this difference was not statistically significant (χ^2^ = 2.10 *p* = 0.15). Among the ETT isolates, resistance was detected for all antibiotics used clinically except for erythromycin, gentamicin and metronidazole (Table S4).

### Microbial community and clinical factors associated with ETT community density

A higher ETT community density at extubation (>10^3^ CFU/cm; *n* = 21/40 [52.50%]) was associated with a higher median (IQR) viable taxonomic richness (5.00 [2.00–8.00] vs. 1.00 [1.00–2.50], *p* = 0.01) and Shannon-Wiener Index (0.79 [0.40–1.32] vs. 0.00 [0.00–0.69], *p* = 0.03) but reduced community evenness (0.56 [0.42–0.93] vs. 1.00 [0.82–1.00], *p* = 0.01) and dominance (0.57 [0.33–0.81] vs. 1.00 [0.50–1.00], *p* = 0.03). Neither the presence of bacterial pathogens (*n* = 11/21 [52.38%] vs. *n* = 5/19 [26.32%]; χ^2^ = 1.84, *p* = 0.18), or their RA when present (median [IQR]: 26.91% [3.62–66.24%] vs. 83.33% [0.54–100.00%], *p* = 0.17) nor the RA of yeast (median [IQR]: 17.17% [3.11–41.10%] vs. 33.80% [5.67–80.00%], *p* = 0.42), detected by extended-quantitative culture, were associated with density. As shown in Table 2, the ETT biofilm community density at extubation was not clearly associated with participants’ clinical characteristics.

**Table 2:** Participant characteristics and their association with an ETT community density >10^3^ CFU/cm of tube segment length (above the cuff*) at extubation

Clinical characteristic	TVC >10^3^ CFU/cm (*n* = 21)	TVC ≤10^3^ CFU/cm (*n* = 19)	*p*-value
Site (Royal Victoria Hospital critical care unit); n (%)	12 (57.14)	5 (26.32)	0.10
Age (years); median (IQR)	65 (50–73)	57 (52.5–68.5)	0.46
Sex (male); n (%)	11 (52.38)	14 (73.68)	0.29
Neurological diagnosis on ICU admission; n (%)	8 (38.10)	4 (21.05)	0.41
Clinical diagnosis of pneumonia on ICU admission; n (%)	5 (23.81)	7 (36.84)	0.58
Respiratory comorbidity; n (%)	5 (23.81)	3 (15.79)	0.70
CCI score; median (IQR)	2.00 (1.00–5.00)	2.00 (1.00–2.50)	0.13
Tracheal intubation duration (days); median (IQR)	2.79 (1.75–6.38)	4.00 (2.63–6.00)	0.64
No. of antibiotics administered during intubation; median (IQR)	2.00 (1.00–3.00)	2.00 (1.00–3.00)	0.67
Diagnosis of VAP during intubation; n (%)	1 (4.76)	4 (21.05)	0.17

Definitions: *TVC* total viable count, *CFU/cm* colony forming units per cm tube segment length, *CCI* Charlson Comorbidity Index, *VAP* ventilator-associated pneumonia

*There was no difference in outcomes when participants were stratified according to the ETT biofilm microbial density below the cuff

## Discussion

In this study we used Illumina MiSeq 16S rRNA marker-gene sequencing and extended-quantitative culture to compare the biofilm community composition and structure on the surface of ETTs above and below the cuff. We identified that a multispecies biofilm community formed, including on the distal end of most participant ETTs, which confirms and expands on prior studies [5, 6, 8, 12, 23]. Subglottic secretion drainage to reduce excessive secretions and controlling the cuff pressure to improve tracheal sealing may reduce the movement of secretions around the ETT cuff and into the lungs [24]. ETT biofilms above and below the cuff were closely related from an ecological perspective based on all measurements made including alpha-diversity metrics, beta-diversity metrics and the density of viable taxa. Therefore, our study results demonstrate that the cuff does not prevent microbial migration from the upper to the lower airways, e.g. due to folds or its shape or material [25–27].

Although the within-participant ETT biofilms were generally spatially homogenous, both culture-independent and -dependent methodologies indicated that the same segments differed between individual participants, as previously reported, which reflects unique patient-level microbiota [13, 14]. It is also known that pathogens found in respiratory secretions may persist in ETT biofilms despite antimicrobial treatment [5–8]. Our findings support this given the congruence between our results (especially using Illumina MiSeq analysis) and that of clinically-directed culture of respiratory secretions, even with the timing of clinical sample collection and extubation often differing. However, genotyping of isolates from different sample types was not possible [6]. Furthermore, neither the presence nor RA of recognised pathogens (extended-quantitative culture) were associated with a higher community density at extubation, but antimicrobial resistance was found among these isolates as demonstrated in prior studies [14]. Antimicrobial resistance phenotypes were also observed in non-pathogenic taxa highlighting that commensal bacteria may be reservoirs of resistance genes [28]. Others reported that additional antimicrobial resistant isolates were more likely in ETT cultures than in clinical samples [23].

In the present study, we observed a greater taxonomic richness via Illumina MiSeq 16S rRNA marker-gene sequencing than by extended-quantitative culture (despite culture-independent internal transcribed spacer [ITS] maker-gene sequencing lacking) which agrees with the findings of others who have compared similar methodologies [14]. The discrepancy may be explained by the detection of taxa by culture-independent methods in a very low RA or of taxa which may be unculturable with the biochemical and biophysical environments provided. It is unclear how long all microorganisms survive on the ETT surface following extubation; however, ETTs were cultured as soon as possible in this study. Biofilms may also harbour microorganisms in a viable but non-culturable state [29]. Conversely, the Illumina MiSeq 16S rRNA marker-gene sequencing procedure used did not exclude DNA from non-viable cells which may affect the community profiling results; however, any impact is expected to be marginal [30].

Many of the taxa detected in the ETT biofilm communities are normal residents of the oral microbiota including obligate anaerobes and streptococci [3], and their presence was associated with greater community diversity, evenness and richness (Illumina MiSeq analysis). Furthermore, *Tetragenococcus* spp. (possible gut bacterium) was found in a higher RA in the segment below the cuff. Although the pathogenic potential of these taxa is uncertain, they may be involved in the initiation of polymicrobial biofilm communities rather than cause clinical decline directly [31]. Moreover, oral commensals have been shown to increase biofilm biomass of recognised pathogens, produce virulence factors, or contribute to the inflammatory response [10, 32, 33]. Of interest, a relatively high proportion of ETT biofilms cultured yeast, namely *Candida* spp., which is in agreement with the results of others [14, 23]. Although its abundance (extended-quantitative culture) was not associated with a higher biofilm community density in this study, *C. albicans* may increase biofilm formation and stability due to hyphal/pseudohyphal structures as evidenced by smaller bacterial cells adhering to both yeast cells and hyphae (Fig. 2C, Arrow 4) [8, 34, 35]. *Candida* airway colonisation may also be a marker of adverse outcomes in patients with VAP e.g. increased mechanical ventilation duration or mortality [36].

Microbial density may be higher in respiratory samples, e.g. tracheal aspirate, compared to the ETT biofilm [9]. This study found a mean ETT biofilm community density of ~10^3^ CFU/cm of tube segment length by culture (up to ~10^6^ CFU/cm; Figure S7) with a community density exceeding the mean associated with increased richness and diversity at extubation. We also determined if there were patient factors which may associate with a higher ETT biofilm community density at extubation. Similar to others, there was no clear relationship between participant characteristics such as age, sex, comorbidities and duration of intubation and the biofilm community density [5, 12].

This study has limitations. The small sample size could introduce bias into the analyses and limits the generalisability of the study findings, particularly to those who are intubated for a longer duration. For example, VAP pathogens such as *Pseudomonas aeruginosa* may dominate biofilm communities during periods of longer intubation and greater antibiotic exposure [3]. The Illumina MiSeq analysis in this study may corroborate this finding (Figure S6), but a larger study is required for confirmation. Nevertheless, to the best of our knowledge, this is the most extensive analysis of biofilm communities forming on segments above and below the ETT cuff. In this study, we did not characterise the mycobiome using culture-independent methods, e.g. ITS1/ITS2 marker-gene sequencing, but this would complement 16S rRNA marker-gene sequencing and enrich community profiling of cross-kingdom biofilms [14]. Although we used SEM to visualise the formation of a biofilm on the participants’ ETT, a detailed assessment of the biofilm topography was not performed. Prior studies reported that certain ETT biofilm characteristics such as the grade or lifecycle stage may be associated with clinical outcomes [5, 37]. Moreover, it is common to link microbiota profiles of airway samples with disease outcomes [38, 39], and in an exploratory analysis we found minimal differences between the ETT biofilm community of the small number of participants who were diagnosed with VAP during intubation compared to those who were not. However, it is important to acknowledge that to investigate the relationship between the ETT biofilm community and prognosis would necessitate following the development of the biofilm over time rather than its characterisation at extubation [12, 40]. We also did not collect participant respiratory secretions at the initiation, during or completion of intubation for a direct community comparison with ETT biofilms [5, 8]. Furthermore, it was observed that ETTs from various manufacturers were used for patient care throughout the study. A clinical trial reported differences in biofilm development between uncoated and silicone-coated polyvinyl chloride ETTs [5]. Whether microbial profiles are impacted by inherent ETT differences, including material composition, was not assessed in this study. Finally, the microbial community profiling encompassed the biofilm on the entire surface of the ETT segment; targeted removal of the biofilm (e.g. via scraping with a scalpel [13]) may be of interest to determine if the intra-ETT microbial community composition and structure differs on the outer and inner luminal surface.

## Conclusions

This prospective observational study of critically ill adults requiring mechanical ventilation with endotracheal intubation demonstrates that the ETT cuff does not provide an effective barrier to microbial migration, as evidenced by conserved biofilm communities forming above and below the cuff. We also observed highly individual ETT biofilm community compositions with potential pathogens, antimicrobial-resistant bacteria and/or yeast present, with the extent of the microbial population density not associated with patient factors. Such heterogeneity should be taken into consideration when designing interventions to reduce biofilm development on the ETT surface; it is unclear if diversity in biofilm communities between patients may impact clinical outcomes.

## Supplementary Information

Additional file 1. Additional details of methods. Additional Figures: Figure S1 Endotracheal tube (ETT) sectioning guide and assessments. (A) Four cross-sectional sections (1-cm lengths labelled A, B, C and D) from the subglottic region above or the distal region below the cuff margin were excised. Sections were divided longitudinally into two pieces for subsequent analyses (eight segments in total). (B) The number of ETTs included and excluded in the subsequent assessments - scanning electron microscopy (SEM), extended-quantitative culture, and Illumina MiSeq 16S rRNA marker-gene sequencing. *Five participants, whose ETT was assessed, were intubated for <3 days. †When extended-quantitative culture of the designated segments (one above ['C'] and one below ['A'] the cuff) was not completed, then the aim was to process these as additional samples for Illumina MiSeq 16S rRNA marker-gene sequencing. Figure S2 Comparison of biofilm community composition in paired (n=54) samples above ('D') and below ('B') the endotracheal cuff. Hierarchical cluster dendrogram showing sample-wise similarity between paired samples (Bray-Curtis dissimilarity based on the variance criterion of the WARD.D2 cluster method). Major community clusters are shown by different coloured boxes. Most endotracheal tubes were processed within 24-hours except for BCH031, BCH032, BCH033, BCH034, BCH035, RVH025 and RVH026 which were stored for 1-5 days prior to processing. Figure S3 Principal coordinate analysis plot comparing microbial communities based on the Euclidean distance metric (adonis analysis [permutational multivariate ANOVA, PERMANOVA]; R2=0.01; p=0.98; 199 (within blocks) permutations; confidence based on 90% confidence interval). Filled circles: blue, community above the endotracheal tube cuff; green, community below the endotracheal tube cuff. Separated communities: The four biofilm communities which appear in the top left-hand quadrant of the plot are of paired segments from two participant endotracheal tubes. There were no obvious differences in clinical characteristics between these participants and the remaining cohort. Figure S4 Comparison of within-participant (n=18) endotracheal tube biofilm community alpha-diversity metrics above ('C' and 'D') and below ('A' and 'B') the cuff. (A) Taxonomic richness [S], (B) community diversity (Shannon-Wiener Index [H]), (C) evenness [Pielou] and (D) dominance [Relative]. In the box and whisker plots, the line inside the box indicates the median and the top and bottom of the box indicates the 25th and 75th percentile, respectively. The whiskers indicate the 90% confidence interval. Matched groups were compared using the Friedman test. Figure S5 Endotracheal tube biofilm community alpha-diversity metrics (microbial richness [observed taxa], diversity [Shannon-Wiener Index], evenness [Pielou], and dominance [relative]) of participants diagnosed with ventilator-associated pneumonia (VAP) during intubation. (A) Comparison of within-participant alpha-diversity metrics above and below the cuff in participants diagnosed with VAP (n=6). (B) Comparison of alpha-diversity metrics between participants with VAP (n=6) and those without VAP (VAP-Free, n=48) in segments above the cuff. (C) Comparison of alpha-diversity metrics between participants with VAP (n=6) and those without VAP (VAP-free, n=48) in segments below the cuff. In the box and whisker plots, the line inside the box indicates the median and the top and bottom of the box indicates the 25th and 75th percentile, respectively. The whiskers indicate the 90% confidence interval. Paired groups were compared using the Wilcoxon-signed rank or Mann-Whitney U test, as appropriate. Figure S6 Relationship between the microbial community composition above and below the endotracheal cuff and selected explanatory variables. An RDA (Redundancy Analysis) biplot on Hellinger-transformed data was used to visualise and test the constrained relationship. Within the plot, the loading of each variable (arrows) and the sample scores (points) are shown. The approximate variance of the variables is indicated by the length of the arrow. The approximate correlation between variables is indicated by the angles between the arrows. Points close together correspond to observations that have similar principal coordinate analysis component scores. Definitions: RVH, Royal Victoria Hospital; BCH, Belfast City Hospital; AbxNo, number of antibiotics administered during intubation; VAPDiag, diagnosis of ventilator-associated pneumonia during intubation; CCI, Charlson Comorbidity Index; TVC, total viable count. Figure S7 Comparison of within-participant (n=40) endotracheal tube biofilm community density (colony-forming units per cm of tube segment length [CFU/cm]) above and below the cuff based on extended-quantitative culture. The geometric mean and 95% confidence interval are shown. Paired groups were compared using the Wilcoxon signed-rank test. Figure S8 Comparison of within-participant (n=40) endotracheal tube biofilm community composition above ('C') and below ('A') the cuff based on extended-quantitative culture (individual isolates were identified by MALDI-ToF, 16S rRNA sequencing or ITS sequencing). (A) Alpha-diversity metrics: taxonomic richness [S], community diversity (Shannon-Wiener Index [H]), evenness [Pielou] and dominance [Relative]. Segments with no growth were excluded resulting in 35 paired ETT segments for analysis. Paired groups were compared using the Wilcoxon signed-rank test. (B) Relative abundance (%) of genera for individual participants. Samples containing empty panels indicate that no microorganisms were recovered ('no growth'). In the box and whisker plots, the line inside the box indicates the median and the top and bottom of the box indicates the 25th and 75th percentile, respectively. The whiskers indicate the 90% confidence interval. Additional Tables: Table S1 Primary diagnoses on intensive care unit admission of individual participants. Table S2 Bacterial and fungal taxa cultured from the endotracheal tube segment biofilm communities. Table S3 Comparison of clinically-directed culture results of respiratory secretions to Illumina MiSeq 16S rRNA marker-gene sequencing and extended-quantitative culture results. Table S4 Summary of antimicrobial resistance amongst selected endotracheal tube biofilm isolates

## Data Availability

Raw sequence data was deposited to the European Nucleotide Archive (Study Accession: PRJEB91743). Other datasets generated and analysed during the current study and the analysis scripts used for microbial community profiling are available from the corresponding author on reasonable request.

## Abbreviations

ASV: Amplicon sequence variant
CCI: Charlson Comorbidity Index
CFU/cm: Colony forming units per cm of tube segment length
ETT: Endotracheal tube
ICU: Intensive care unit
ITS: Internal transcribed spacer
PBS: Phosphate buffered saline
QSRS: Quarter-strength Ringers Solution
RA: Relative abundance
SEM: Scanning electron microscopy
TVC: Total viable count
VAP: Ventilator-associated pneumonia
