# Supplementary material for: Biofilm communities above and below the cuff of endotracheal tubes are spatially homogenous
Source: Respir Res. 2026 Jan 22;27:74. doi: 10.1186/s12931-025-03485-2 (PMC12911081; doi:10.1186/s12931-025-03485-2)
Supplement: Supplementary file 1 — Additional file 1. Additional details of methods. Additional Figures: Figure S1 Endotracheal tube (ETT) sectioning guide and assessments. (A) Four cross-sectional sections (1-cm lengths labelled A, B, C and D) from the subglottic region above or the distal region below the cuff margin were excised. Sections were divided longitudinally into two pieces for subsequent analyses (eight segments in total). (B) The number of ETTs included and excluded in the subsequent assessments - scanning electron microscopy (SEM), extended-quantitative culture, and Illumina MiSeq 16S rRNA marker-gene sequencing. *Five participants, whose ETT was assessed, were intubated for <3 days. †When extended-quantitative culture of the designated segments (one above ['C'] and one below ['A'] the cuff) was not completed, then the aim was to process these as additional samples for Illumina MiSeq 16S rRNA marker-gene sequencing. Figure S2 Comparison of biofilm community composition in paired (n=54) samples above ('D') and below ('B') the endotracheal cuff. Hierarchical cluster dendrogram showing sample-wise similarity between paired samples (Bray-Curtis dissimilarity based on the variance criterion of the WARD.D2 cluster method). Major community clusters are shown by different coloured boxes. Most endotracheal tubes were processed within 24-hours except for BCH031, BCH032, BCH033, BCH034, BCH035, RVH025 and RVH026 which were stored for 1-5 days prior to processing. Figure S3 Principal coordinate analysis plot comparing microbial communities based on the Euclidean distance metric (adonis analysis [permutational multivariate ANOVA, PERMANOVA]; R2=0.01; p=0.98; 199 (within blocks) permutations; confidence based on 90% confidence interval). Filled circles: blue, community above the endotracheal tube cuff; green, community below the endotracheal tube cuff. Separated communities: The four biofilm communities which appear in the top left-hand quadrant of the plot are of paired segments from two participant [file 12931_2025_3485_MOESM1_ESM.docx]

**Supplementary Materials**

**Title: Biofilm communities above and below the cuff of endotracheal tubes are spatially homogenous**

Gisli G. Einarsson,^1^ Sujata Das,^1^ Jonathan A. Silversides,^2,3^ Nerielle Fundano,^3^ Elliott Lonsdale,^3^ Ronan McMullan,^2^ Daniel F. McAuley,^2,3^ Nicola J. Irwin,^1^ Colin P. McCoy,^1^ Matthew P. Wylie,^1^ Laura J. Sherrard.^1^

^1^School of Pharmacy, Queen’s University Belfast, Belfast, United Kingdom.

^2^Wellcome-Wolfson Institute for Experimental Medicine, Queen’s University Belfast, Belfast, United Kingdom.

^3^Department of Critical Care, Belfast Health and Social Care Trust, Belfast, United Kingdom.

**Additional Methods**

**Scanning electron microscopy**

Segments fixed with the glutaraldehyde solution were washed in sterile phosphate buffered saline three times for 1-hour and then progressively dehydrated in a graded series (30%, 50%, 70%, 90%) of ethanol dilutions (in sterile water) for 15-minutes each and finally in 100% ethanol for 1-hour at room temperature. Samples were dried for 24-hours in a fume cupboard, mounted on aluminum stubs using adhesive carbon tape and sputter coated with a thin Au film (~15-20 nm) (1).

**Biofilm disruption from the endotracheal tube surface**

Each endotracheal tube (ETT) section excised was gently washed by dipping in sterile quarter-strength Ringers Solution (QSRS) to remove poorly adherent material. Sections were then divided longitudinally, using a sterile scalpel, into two pieces for subsequent analyses (eight segments in total). For Illumina MiSeq 16S rRNA marker-gene sequencing and extended-quantitative culture, each segment was transferred to 10 mL sterile QSRS and the biofilm was dislodged from the ETT surface using a vortexing-sonication-vortexing method as follows: the segments were vortexed at full speed for ~1-minute, sonicated in an ultrasonic bath (Branson 3510E-MT) for 10-minutes (40 kHz) and then vortexed for ~1 minute once more (2). The sonicate was concentrated by centrifugation at 3000 g for 10-minutes. The supernatant (9 mL) was carefully aspirated, and the sonicate was resuspended in the remaining 1 mL. The sonicate suspension was processed by culture immediately. The sonicates for Illumina MiSeq 16S rRNA marker-gene sequencing were stored at -80°C until processed.

**Illumina MiSeq 16S rRNA marker-gene sequencing and analysis**

Illumina MiSeq 16S rRNA marker-gene sequencing was performed according to a standard Illumina workflow, except for using custom sequencing primers from the Earth Microbiome protocol (<https://earthmicrobiome.org/protocols-and-standards/16s/>) (3). Briefly, extraction of gDNA was performed on the MagNA Pure DNA extraction system (Roche Diagnostics), followed by library preparation and sequencing on the Illumina MiSeq. Positive and negative (QSRS) controls were included throughout the DNA extraction, library preparation and sequencing runs. The identities of all bacterial taxa were reported based on the V4 hypervariable region.

***gDNA extraction***

Prior to loading the samples onto the Illumina MiSeq sequencer, polymerase chain reaction (PCR) products were cleaned using the AxyPrep Mag PCR Clean-up kit. Briefly, 35 µL of vortexed magnetic beads were aliquoted with the PCR product into a 96-well plate, mixed, and incubated at room temperature for 5-minutes. Beads were separated using a magnetic stand, and the supernatant was discarded. Beads were washed twice with 180 µL of 70% ethanol, air-dried for ≤5-minutes, and eluted in 50 µL diethyl pyrocarbonate-treated water. Cleaned products were quantified using the Quant-iT™ PicoGreen® dsDNA Assay Kit (Life Technologies, UK). Equimolar amounts (≤20 µL per sample) were pooled, with failed reactions added at fixed volumes. Final quantification (triplicate PicoGreen® assay) was performed before storage at –20°C/–80°C for Illumina MiSeq 16S rRNA sequencing.

***Data processing***

Raw paired-end 16S rRNA sequences were processed using the DADA2 pipeline (v1.22) in R (v4.1.2; <https://www.r-project.org/>) to infer high-resolution amplicon sequence variants (ASVs). Forward and reverse reads were quality-filtered and trimmed using filterAndTrim() with parameters truncLen = c(250, 220), trimLeft = 20 (to remove primers), maxN = 0, and maxEE = c(2,2). Reads were dereplicated (derepFastq()) and error rates were learned separately for forward and reverse reads (learnErrors()). Denoising was performed using the core dada() algorithm and paired-end reads were merged (mergePairs()) with a minimum overlap of 12 bp and no mismatches allowed in the overlap region. Chimeric sequences were removed via removeBimeraDenovo() (method = "consensus"), retaining 98% (calculated as sum(seqtab.nochim)/sum(seqtab)) of non-chimeric reads. Taxonomy was assigned using the SILVA v138 reference database (assignTaxonomy()) with species-level resolution. For phylogenetic analysis, ASV sequences were aligned with DECIPHER (AlignSeqs()), converted to a phyDat object, and used to construct a maximum-likelihood tree using phangorn (optim.pml()). The final ASV table, taxonomy, and tree were combined into a phyloseq object for downstream analysis. Alpha-diversity and beta-diversity metrics were computed using the microbiome and vegan packages, with statistical testing (e.g., PERMANOVA) performed where applicable.

***Negative controls and putative contaminant identification and removal***

Potential contaminants were identified and removed using the *decontam* R package (v1.14.0; (4)) applied to the phyloseq object containing all samples, including negative controls. The *prevalence-based* method was used, which compares the occurrence frequency of each ASV in true samples versus negative controls via Fisher’s exact test to identify taxa disproportionately represented in controls. Negative controls were flagged in the sample metadata (column "SampleType" = NegativeControl"). Due to unavailable DNA concentration data, the *frequency-based* contaminant detection method was not used at this stage, although the framework was retained to enable re-analysis once DNA quantification data become available. The initial list of putative contaminants was visually inspected using bar plots stratified by sample type to confirm likely contamination patterns. Taxa known to be common kit or reagent contaminants were subsequently removed including: *Corynebacterium* ASV12, *Micrococcus* ASV25, *Pelomonas* ASV54, *Novosphingobium* ASV94, *Achromobacter* ASV108, *Comamonas* ASV127, *Gardnerella* ASV132, *Aridibacter* ASV136, *Nocardioides* ASV154, *Galbitalea* ASV169, *Mucilaginibacter* ASV183, *Tenacibaculum* ASV201, *Streptomyces* ASV202, *Rubrobacter* ASV288, and *Allobaculum* ASV302. Following contaminant removal, ASVs were re-pruned from the phyloseq object, and relative abundances were re-normalized to sequencing depth (total-sum scaling) prior to downstream analyses (e.g., ordination and diversity). This ensured that compositional data reflected the decontaminated microbial community structure in each sample.

***Normalisation***

For count data (ASVs), segments were normalised to 10,000 amplicons/sample based on rarefaction analysis.

**Extended-quantitative culture**

When extended-quantitative culture was performed (ETTs processed within 24-hours of extubation), processing steps were completed within an anaerobic cabinet (80% N_2_, 10% H_2_, 10% CO_2_) when feasible. Briefly, after 10-fold serial dilution of two segment suspensions (one above and one below the cuff), 100 μL aliquots (including the neat) were spread onto different types of agar which were incubated either anaerobically (anaerobic blood agar, kanamycin-vancomycin laked blood agar), aerobically (horse blood agar, Mannitol salt red violet agar, MacConkey agar and Sabouraud Dextrose agar), or in 5% CO_2_ (chocolate blood agar) at 37°C for 48 hours except for anaerobic cultures (incubated for 5-7 days). All distinct colony morphologies were enumerated and pure cultures were identified to the species-level, if possible, using matrix-assisted laser desorption-ionization-time of flight mass spectrometry (Bruker MALDI Biotyper®) or near-full length 16S rRNA gene sequencing (bacteria) or internal transcribed spacer (ITS-1 and ITS-4) region sequencing (fungi) (5-8). The estimated density (total viable count) of the biofilm community of each segment was based on the combined count of unique taxa identified and expressed as colony forming units per cm of tube segment length (CFU/cm). The limit of detection was 10 CFU/cm.

**Further details of measurements and data analyses**

Alpha-diversity metrics were calculated - taxonomic richness [S], Shannon-Wiener Index [H], evenness [Pielou] and dominance [Relative]. For beta-diversity analysis (Illumina MiSeq dataset), count data were Hellinger-transformed, and the Bray-Curtis quantitative index of dissimilarity was used to compare community structures. The Hellinger transformation reduces the influence of sequencing depth, accounts for compositionality and dominant taxa, while Bray-Curtis quantifies compositional differences based on species abundances.

To determine the optimal number of clusters in the microbial community data (**Figure S2**), a silhouette analysis was performed on a Bray-Curtis distance matrix derived from relative abundance-transformed ASV counts using the cluster (v2.1.2) and factorextra (v1.0.7) packages in R. A distance matrix was then computed using Bray-Curtis dissimilarity. Next, partitioning around medoids (PAM) clustering was applied for cluster numbers (k) ranging from 2 to 10, and the average silhouette width was calculated for each k. The optimal cluster number was identified as the k with the highest average silhouette width (k = 6) (9).

Principal coordinate analysis was performed (**Figure S3**) to compare beta-diversity differences between ETT segments (above and below the cuff) using PERMANOVA (adonis2 function, vegan package v2.5-7). To account for paired sampling within individuals, permutations were constrained within each subject using a within-block design implemented via the how(blocks = Subject) function. This approach tests for within-patient differences while controlling for inter-patient variation ensuring that any detected differences reflect within- rather than between-individual variation.

A redundancy analysis (RDA) was conducted (**Figure S6**) using the microViz package in R to examine relationships between microbial community composition (genus-level) and selected variables. Genus abundance was Hellinger-transformed to address compositional bias prior to analysis. The RDA model incorporated 10 constraining variables with both microbial diversity metrics (taxonomic richness [S], Shannon-Wiener Index [H], evenness [Pielou] and dominance [Relative]) and clinical parameters (antibiotic exposure, intubation time etc.) represented. The ordination results were visualised by plotting samples coloured by tube section (above or below the cuff) and shaped by intensive care unit site, while highlighting the top 10 influential genera and constraint vectors. The approximate variance of the variables is indicated by the length of the arrow and the approximate correlation between variables is indicated by the angles between those arrows. The strength of the correlations was further examined with Spearman’s rank correlation coefficient with a Benjamini-Hochberg adjustment for multiple testing.

**Figures**

**A B**


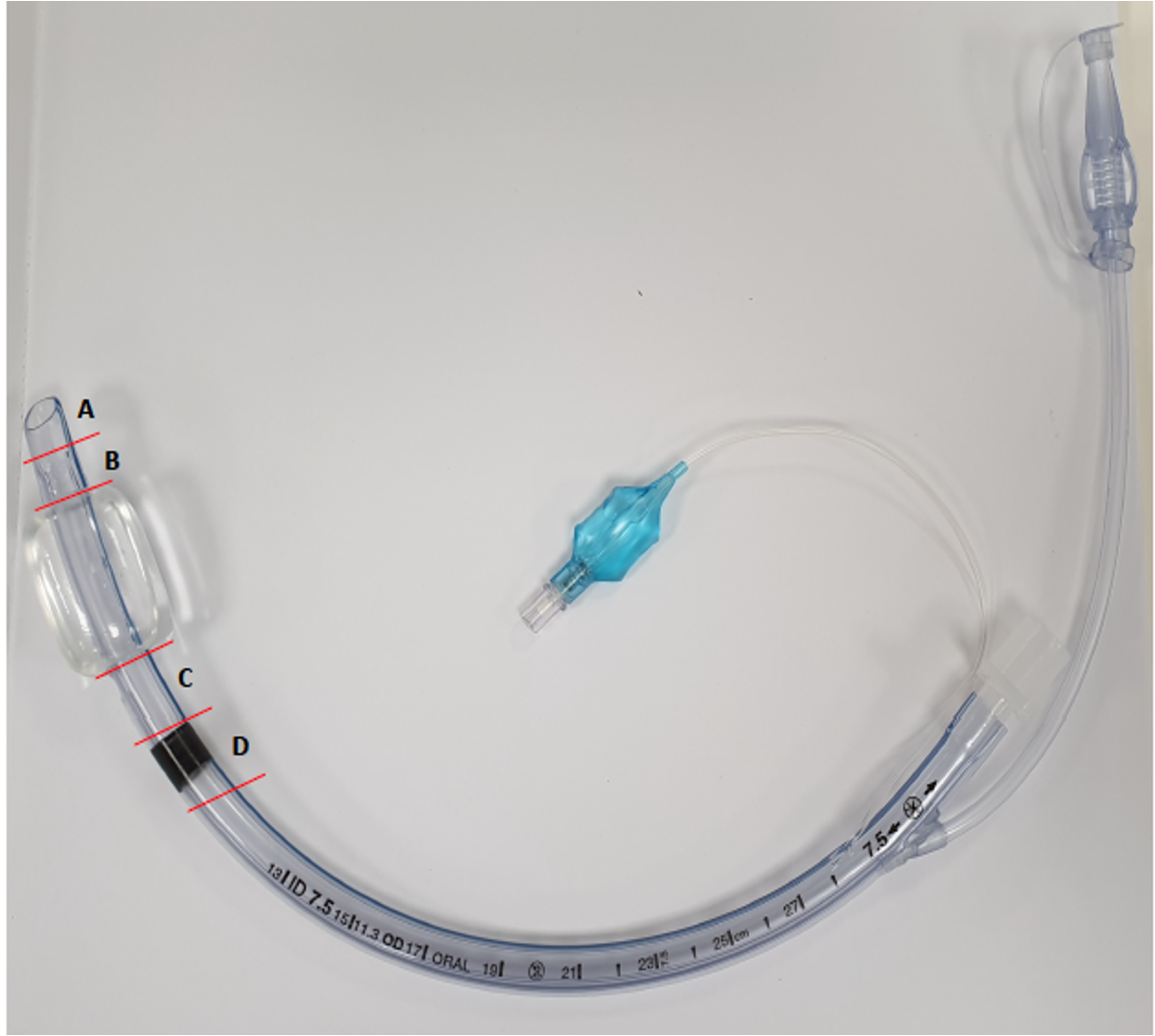

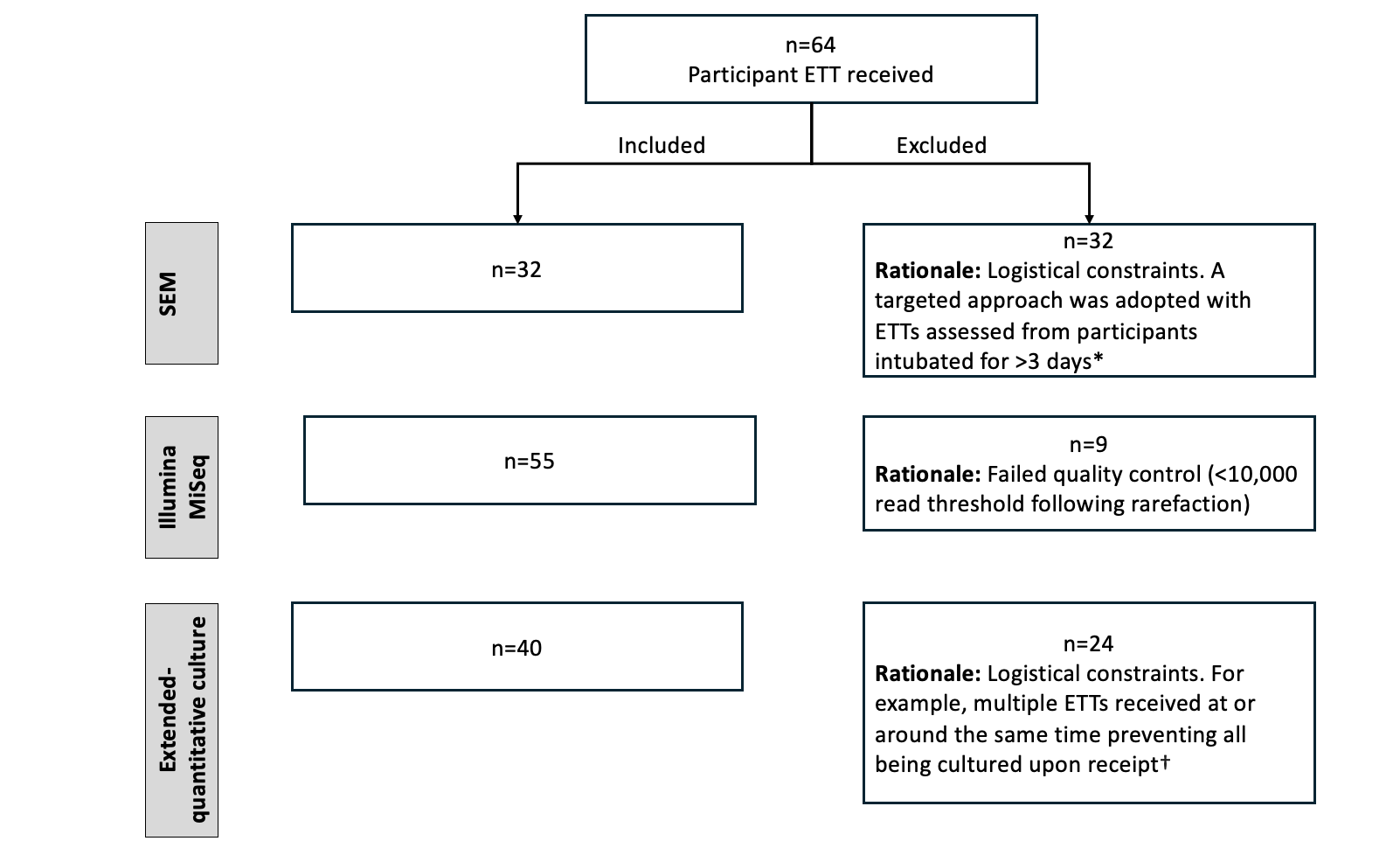


**Figure S1** Endotracheal tube (ETT) sectioning guide and assessments. **(A)** Four cross-sectional sections (1-cm lengths labelled A, B, C and D) from the subglottic region above or the distal region below the cuff margin were excised. Sections were divided longitudinally into two pieces for subsequent analyses (eight segments in total). **(B)** The number of ETTs included and excluded in the subsequent assessments - scanning electron microscopy (SEM), extended-quantitative culture, and Illumina MiSeq 16S rRNA marker-gene sequencing.

*Five participants, whose ETT was assessed, were intubated for <3 days. †When extended-quantitative culture of the designated segments (one above [‘C’] and one below [‘A’] the cuff) was not completed, then the aim was to process these as additional samples for Illumina MiSeq 16S rRNA marker-gene sequencing.


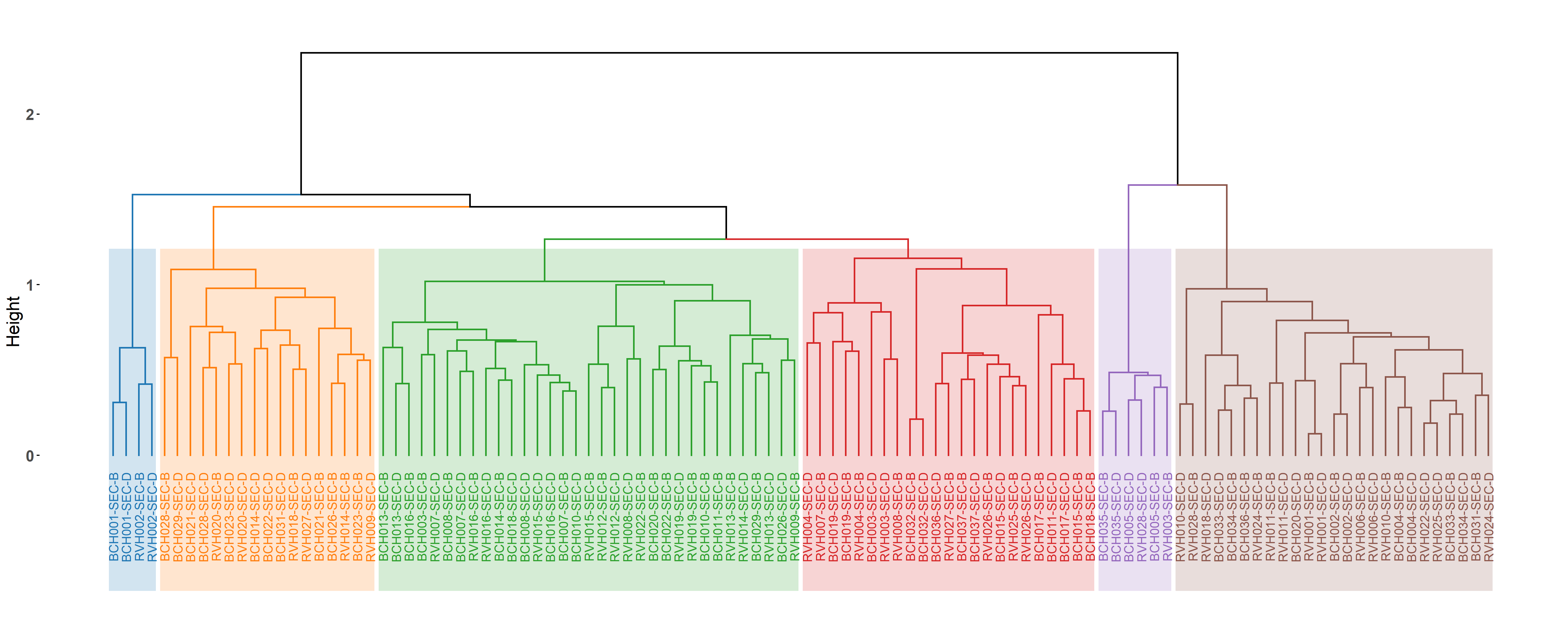


**Figure S2** Comparison of biofilm community composition in paired (n=54) samples above (‘D’) and below (‘B’) the endotracheal cuff. Hierarchical cluster dendrogram showing sample-wise similarity between paired samples (Bray–Curtis dissimilarity based on the variance criterion of the WARD.D2 cluster method). Major community clusters are shown by different coloured boxes. Most endotracheal tubes were processed within 24-hours except for BCH031, BCH032, BCH033, BCH034, BCH035, RVH025 and RVH026 which were stored for 1-5 days prior to processing.


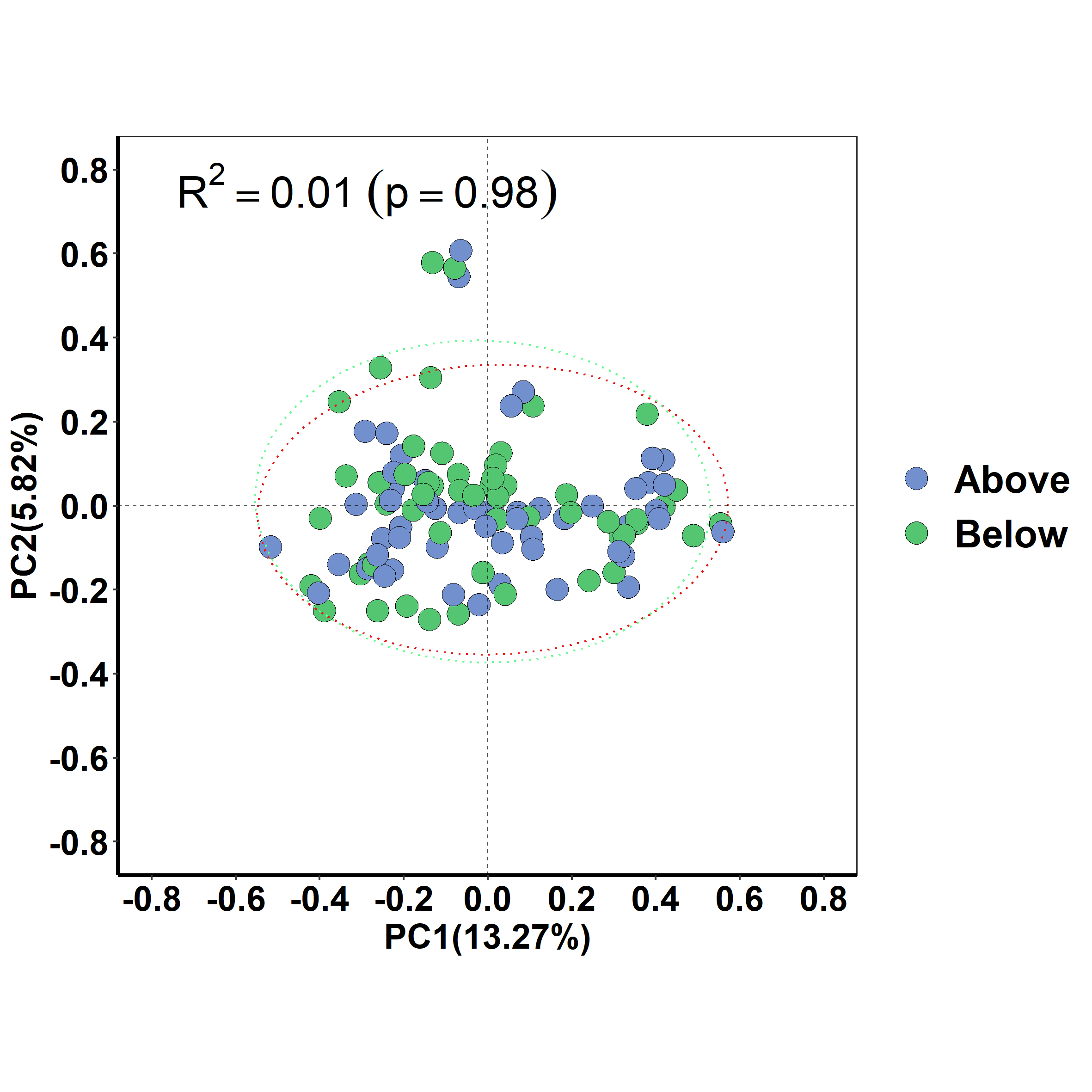
**Figure S3** Principal coordinate analysis plot comparing microbial communities based on the Euclidean distance metric (adonis analysis [permutational multivariate ANOVA, PERMANOVA]; R^2^=0.01; p=0.98; 199 (within block) permutations; confidence based on 90% confidence interval). Filled circles: blue, community above the endotracheal tube cuff; green, community below the endotracheal tube cuff.

*Separated communities*: The four biofilm communities which appear in the top left-hand quadrant of the plot are of paired segments from two participant endotracheal tubes (BCH001 and RVH002; dominated by Escherichia-Shigella). There were no obvious differences in clinical characteristics between these participants and the remaining cohort.


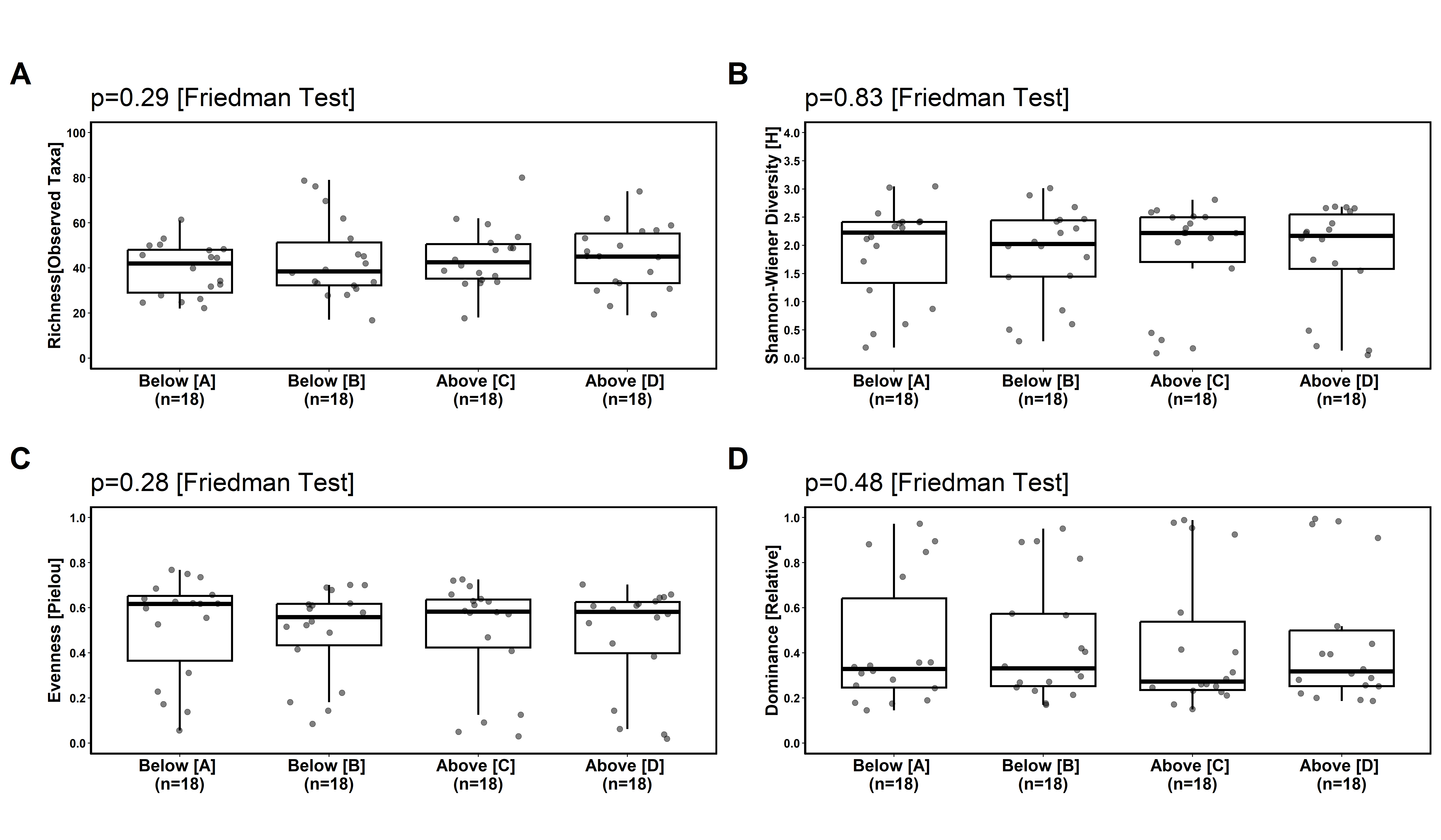


**Figure S4** Comparison of within-participant (n=18) endotracheal tube biofilm community alpha-diversity metrics above (‘C’ and ‘D’) and below (‘A’ and ‘B’) the cuff. **(A)** Taxonomic richness [S], **(B)** community diversity (Shannon Wiener Index [H]), **(C)** evenness [Pielou] and **(D)** dominance [Relative]. In the box and whisker plots, the line inside the box indicates the median and the top and bottom of the box indicates the 25^th^ and 75^th^ percentile, respectively. The whiskers indicate the 90% confidence interval. Matched groups were compared using the Friedman test.

**
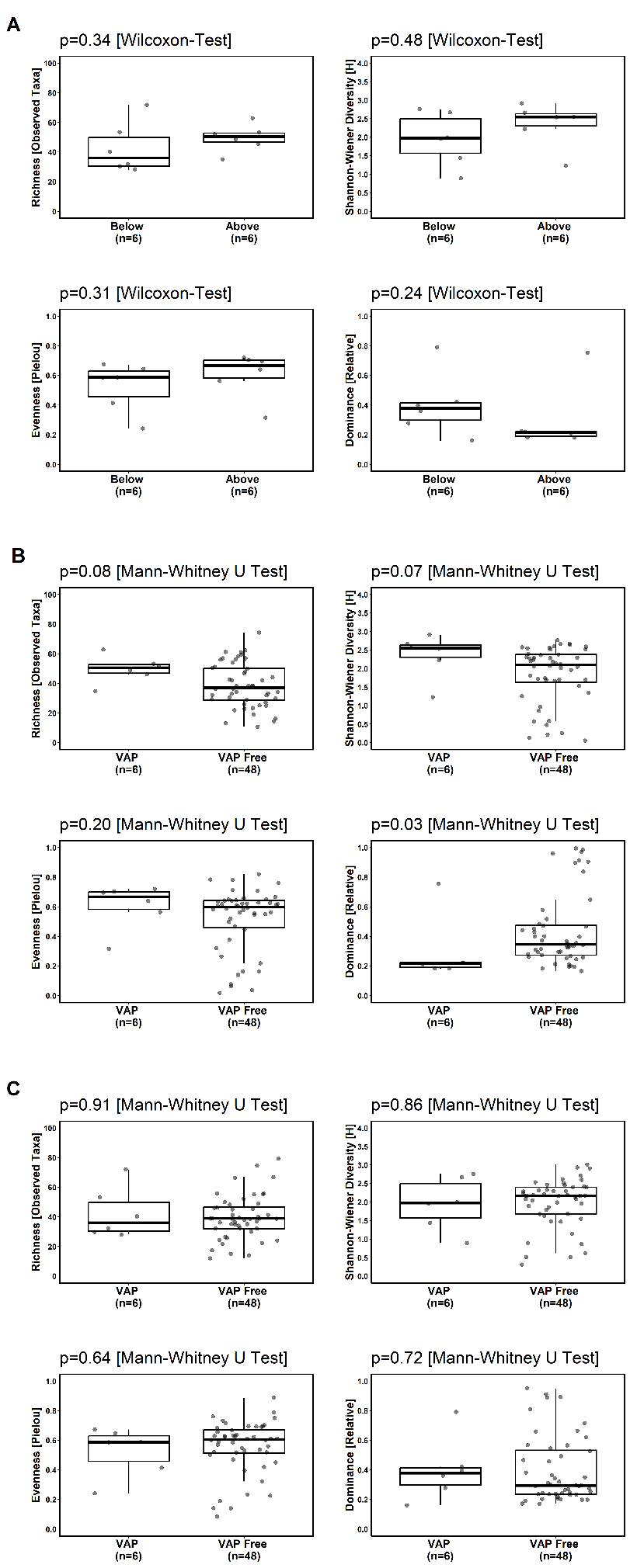
**

**Figure S5 Endotracheal tube biofilm community alpha-diversity metrics (**microbial richness [observed taxa], diversity [Shannon–Wiener Index], evenness [Pielou], and dominance [relative]) **of participants diagnosed with ventilator-associated pneumonia (VAP) during intubation. (A)** Comparison of within-participant alpha-diversity metrics **above** and **below the cuff** in participants diagnosed with VAP (n=6). **(B)** Comparison of alpha-diversity metrics between participants **with VAP (n=6)** and those **without VAP (VAP-free, n=48)** in segments **above the cuff**. **(C)** Comparison of alpha-diversity metrics between participants **with VAP (n=6)** and those **without VAP (VAP-free, n=48)** in segments **below the cuff**. In the box and whisker plots, the line inside the box indicates the median and the top and bottom of the box indicates the 25^th^ and 75^th^ percentile, respectively. The whiskers indicate the 90% confidence interval. Paired groups were compared using the Wilcoxon-signed rank or **Mann-Whitney U test, as appropriate**.

**
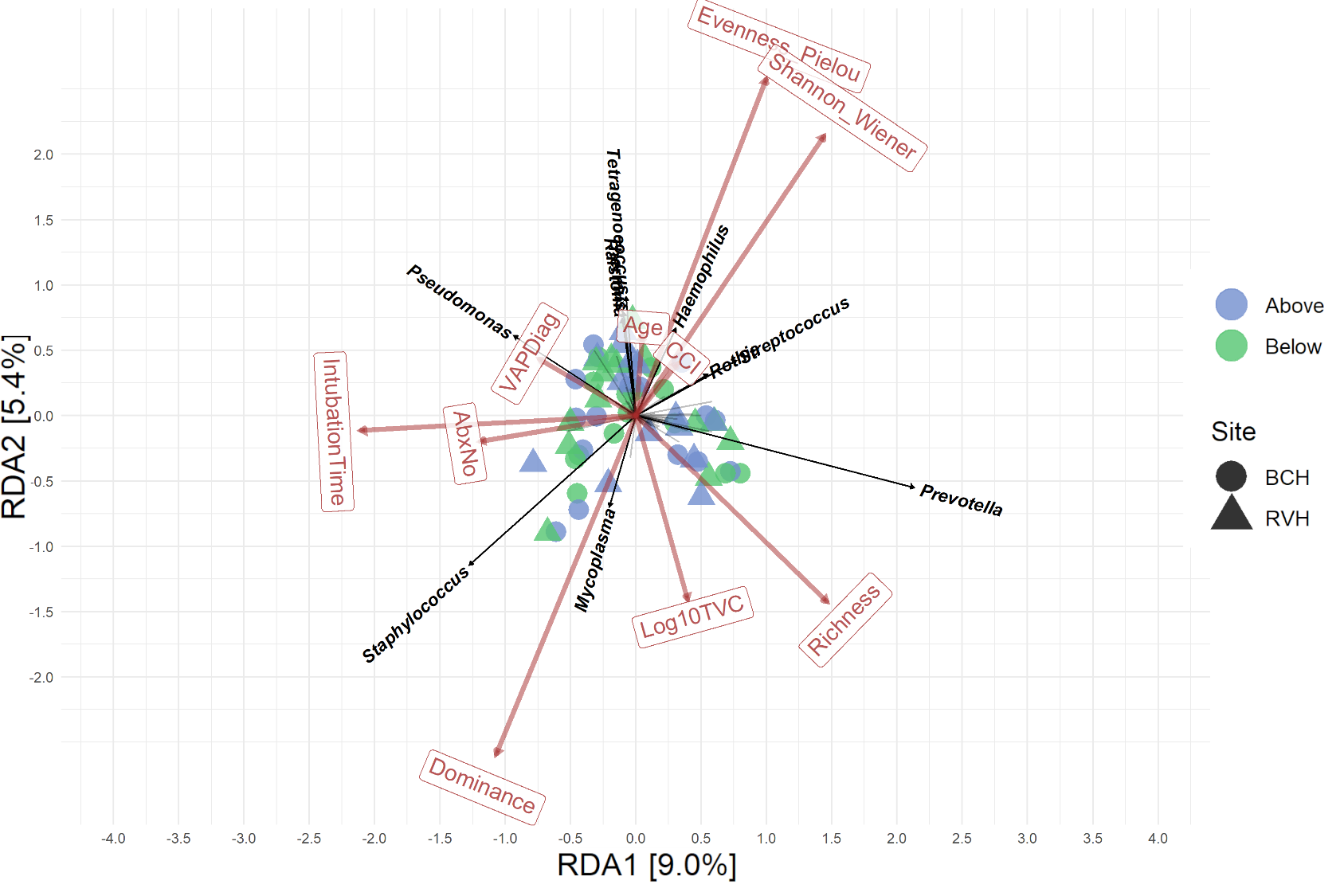
**

**Figure S6** Relationship between the microbial community composition above and below the endotracheal cuff and selected explanatory variables. An RDA (Redundancy Analysis) biplot on Hellinger-transformed data was used to visualise and test the constrained relationship. Within the plot, the loading of each variable (arrows) and the sample scores (points) are shown. The approximate variance of the variables is indicated by the length of the arrow. The approximate correlation between variables is indicated by the angles between the arrows. Points close together correspond to observations that have similar principal coordinate analysis component scores.

*Definitions:* RVH, Royal Victoria Hospital; BCH, Belfast City Hospital; AbxNo, number of antibiotics administered during intubation; VAPDiag, diagnosis of ventilator-associated pneumonia during intubation; CCI, Charlson Comorbidity Index; TVC, total viable count.


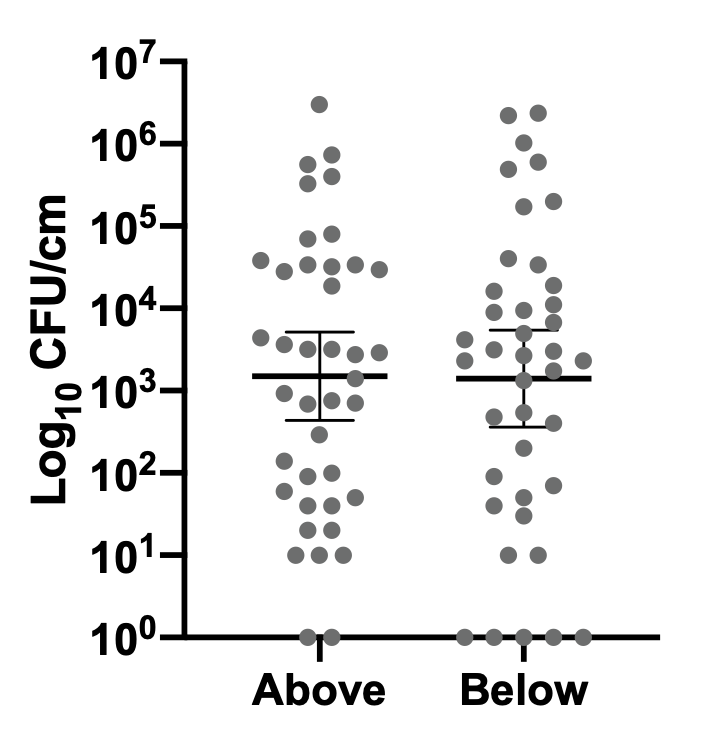


**Figure S7** Comparison of within-participant (n=40) endotracheal tube biofilm community density (colony-forming units per cm of tube segment length [CFU/cm]) above and below the cuff based on extended-quantitative culture. The geometric mean and 95% confidence interval are shown. Paired groups were compared using the Wilcoxon signed-rank test.

**
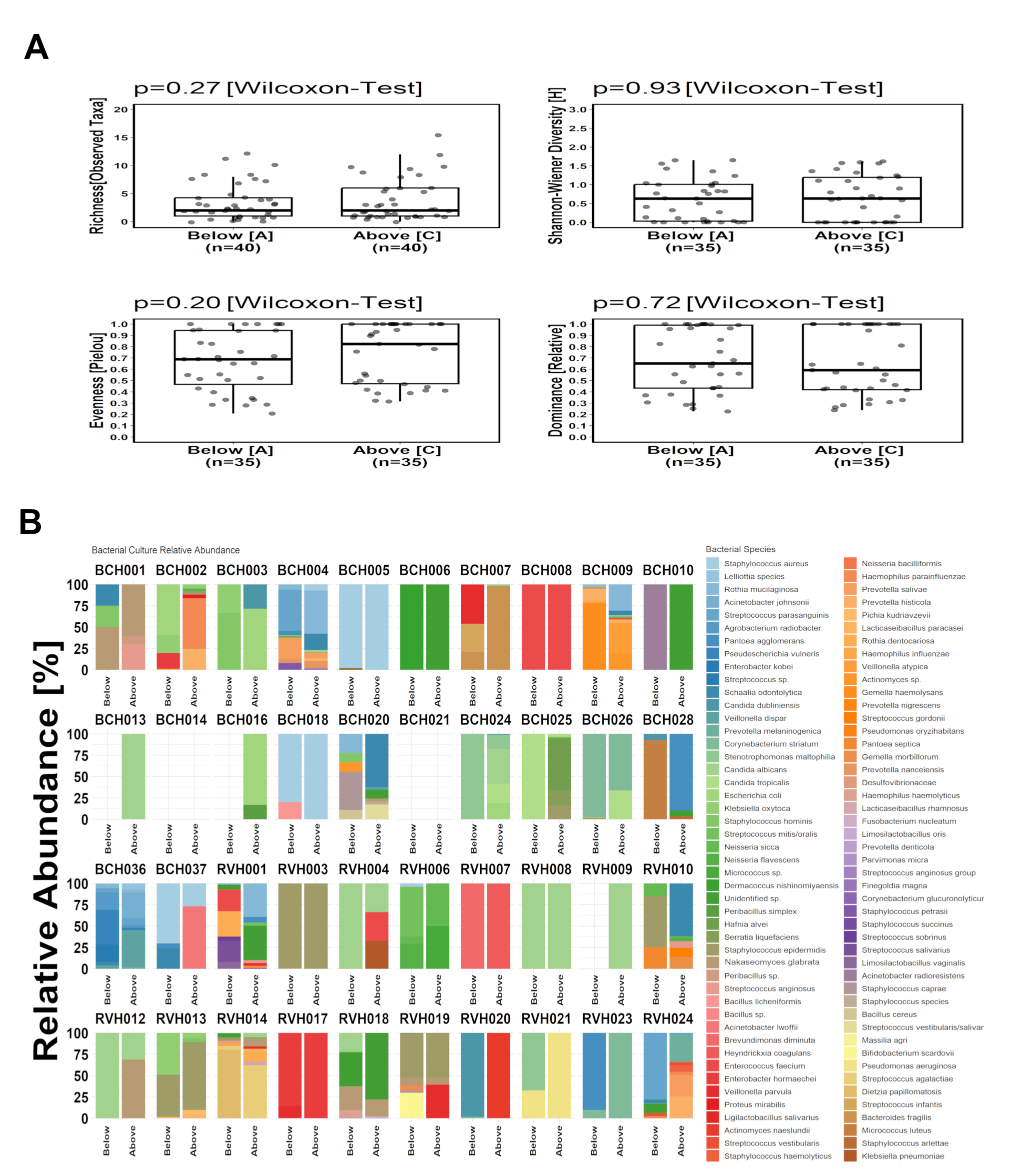
**

**Figure S8** Comparison of within-participant (n=40) endotracheal tube biofilm community composition above (‘C’) and below (‘A’) the cuff based on extended-quantitative culture (individual isolates were identified by MALDI-ToF, 16S rRNA sequencing or ITS sequencing). **(A)** Alpha-diversity metrics: taxonomic richness [S], community diversity (Shannon Wiener Index [H]), evenness [Pielou] and dominance [Relative]. Segments with no growth were excluded resulting in 35 paired ETT segments for analysis. Paired groups were compared using the Wilcoxon signed-rank test. **(B)** Relative abundance (%) of genera for individual participants. Samples containing empty panels indicate that no microorganisms were recovered (‘no growth’). In the box and whisker plots, the line inside the box indicates the median and the top and bottom of the box indicates the 25^th^ and 75^th^ percentile, respectively. The whiskers indicate the 90% confidence interval.

**Tables**

**Table S1** Primary diagnoses on intensive care unit admission of individual participants.

| **Primary diagnosis** | **Category** |
| --- | --- |
| Perforated duodenal ulcer; Peritonitis | Gastrointestinal |
| Post-ictal state; Aspiration pneumonia | Neurological |
| Polytrauma: Intracerebral haemorrhage, multiple rib fractures, tension haemopneumothorax | Neurological |
| Aneurysmal subarachnoid haemorrhage complicated by right internal carotid artery occlusion requiring stent placement | Neurological |
| Out of hospital cardiac arrest secondary to anterior myocardial infarction & subsequent aspiration pneumonia | Cardiovascular |
| Community-acquired pneumonia | Respiratory |
| Acute left ventricular impairment | Cardiovascular |
| Central line infection and pneumonia | Respiratory |
| Small bowel ischaemia secondary to superior mesenteric artery occlusion | Gastrointestinal |
| Seizure | Neurological |
| Postoperative pulmonary complications following elective thoracotomy | Respiratory |
| Out of hospital cardiac arrest secondary to anterior myocardial infarction & subsequent aspiration pneumonia | Cardiovascular |
| Mixed overdose & aspiration pneumonia | Other |
| Status epilepticus | Neurological |
| Status epilepticus; Aspiration pneumonia | Neurological |
| Presumed cerebrovascular accident | Neurological |
| Postoperative pulmonary complications following elective oesophagectomy | Respiratory |
| Biliary tract sepsis | Gastrointestinal |
| CNS lymphoma and hydrocephalus | Neurological |
| Stroke | Neurological |
| Haemorrhagic shock due to intercostal artery injury associated with rib fractures | Respiratory |
| Small bowel obstruction | Gastrointestinal |
| Subdural haematoma; Multiple rib fractures | Neurological |
| Subdural and intraparenchymal haemorrhage; Aspiration pneumonia | Neurological |
| Overdose; Aspiration pneumonia | Other |
| Peritonitis due to anastomotic leak | Gastrointestinal |
| Hospital acquired pneumonia | Respiratory |
| Acute cholangitis | Gastrointestinal |
| Community acquired pneumonia | Respiratory |
| Mixed overdose | Other |
| Post operative ileus following cystoprostatectomy and ileal conduit | Gastrointestinal |
| Post-operative oesophagectomy | Gastrointestinal |
| Status epilepticus; aspiration pneumonia | Neurological |
| Post-operative oesophagectomy | Gastrointestinal |
| Potential Guillain-Barre Syndrome | Neurological |
| Acute exacerbation of asthma | Respiratory |
| Intracerebral haemorrhage secondary to posterior inferior cerebellar artery aneurysm rupture | Neurological |
| Out of hospital cardiac arrest felt secondary to primary arrhythmia or heart failure | Cardiovascular |
| Pneumococcal community acquired pneumonia; Sepsis | Respiratory |
| Community acquired pneumonia | Respiratory |
| Community acquired pneumonia; Severe biventricular heart failure | Respiratory |
| Perforated sigmoid colon | Gastrointestinal |
| Group A beta haemolytic strep bacteraemia initially from leg cellulitis; Hospital-acquired pneumonia | Other |
| Post-operative oesophagectomy | Gastrointestinal |
| Small bowel obstruction | Gastrointestinal |
| Community acquired pneumonia | Respiratory |
| Perforated duodenal ulcer | Gastrointestinal |
| Paraneoplastic syndrome | Neurological |
| Mixed overdose; Aspiration pneumonia | Neurological |
| Bladder perforation | Other |
| Polytrauma | Other |
| Post-operative gastrointestinal stromal tumour resection | Gastrointestinal |
| Hospital acquired pneumonia | Respiratory |
| Seizure | Neurological |
| Myocardial infarction; Left ventricular failure; Cardiac arrest | Cardiovascular |
| Status epilepticus | Neurological |
| Diverticular perforation and infected aortic graft | Gastrointestinal |
| Mixed overdose; Aspiration pneumonia | Neurological |
| Anaphylaxis | Other |
| Polytrauma | Gastrointestinal |
| Post-operative oesophagectomy | Gastrointestinal |
| Out of Hospital cardiac arrest; Anterior ST-segment elevation myocardial infarction | Cardiovascular |
| Post-operative posterior fossa craniotomy and excision of cerebellar tumour | Neurological |
| Variceal upper gastrointestinal bleeding; Subarachnoid haemorrhage | Gastrointestinal |

**Table S2** Bacterial and fungal taxa cultured from the endotracheal tube segment biofilm communities.

| Identity | Count  (individual taxa) |
| --- | --- |
| *Acinetobacter johnsonii* | 1 |
| *Acinetobacter lwoffii* | 1 |
| *Acinetobacter radioresistens* | 1 |
| *Actinomyces naeslundii* | 1 |
| *Actinomyces* spp. | 2 |
| *Agrobacterium radiobacter* | 2 |
| *Bacillus cereus* | 1 |
| *Bacillus licheniformis* | 2 |
| *Bacillus* spp. | 1 |
| *Bacteroides fragilis* | 2 |
| *Bifidobacterium scardovii* | 1 |
| *Brevundimonas diminuta* | 1 |
| *Candida albicans* | 18 |
| *Candida dubliniensis* | 5 |
| *Candida tropicalis* | 5 |
| *Corynebacterium glucuronolyticum* | 1 |
| *Corynebacterium striatum* | 5 |
| *Dermacoccus nishinomiyaensis* | 1 |
| *Desulfovibrionaceae* | 1 |
| *Dietzia papillomatosis* | 1 |
| *Enterobacter cloacae complex* | 5 |
| *Enterococcus faecium* | 5 |
| *Escherichia coli* | 6 |
| *Finegoldia magna* | 1 |
| *Fusobacterium nucleatum* | 1 |
| *Gemella haemolysans* | 1 |
| *Gemella morbillorum* | 1 |
| *Haemophilus haemolyticus* | 1 |
| *Haemophilus influenzae* | 3 |
| *Haemophilus parainfluenzae* | 7 |
| *Hafnia alvei* | 1 |
| *Heyndrickxia coagulans* | 2 |
| *Klebsiella oxytoca* | 5 |
| *Klebsiella pneumoniae* | 1 |
| *Lacticaseibacillus paracasei* | 2 |
| *Lacticaseibacillus rhamnosus* | 3 |
| *Lelliottia* spp. | 1 |
| *Ligilactobacillus salivarius* | 1 |
| *Limosilactobacillus oris* | 1 |
| *Limosilactobacillus vaginalis* | 1 |
| *Massilia agri* | 1 |
| *Micrococcus luteus* | 1 |
| *Micrococcus* spp. | 1 |
| *Nakaseomyces glabrata* | 12 |
| *Neisseria bacilliformis* | 1 |
| *Neisseria flavescens* | 6 |
| *Neisseria sicca* | 5 |
| *Pantoea agglomerans* | 8 |
| *Pantoea septica* | 1 |
| *Parvimonas micra* | 1 |
| *Peribacillus simplex* | 1 |
| *Peribacillus* spp. | 1 |
| *Pichia kudriavzevii* | 2 |
| *Prevotella denticola* | 1 |
| *Prevotella histicola* | 7 |
| *Prevotella melaninogenica* | 2 |
| *Prevotella nanceiensis* | 3 |
| *Prevotella nigrescens* | 1 |
| *Prevotella salivae* | 3 |
| *Proteus mirabilis* | 2 |
| *Pseudescherichia vulneris* | 1 |
| *Pseudomonas aeruginosa* | 2 |
| *Pseudomonas oryzihabitans* | 2 |
| *Rothia dentocariosa* | 3 |
| *Rothia mucilaginosa* | 9 |
| *Schaalia odontolytica* | 9 |
| *Serratia liquefaciens* | 1 |
| *Staphylococcus arlettae* | 1 |
| *Staphylococcus aureus* | 11 |
| *Staphylococcus caprae* | 2 |
| *Staphylococcus epidermidis* | 16 |
| *Staphylococcus haemolyticus* | 1 |
| *Staphylococcus hominis* | 5 |
| *Staphylococcus petrasii* | 1 |
| *Staphylococcus* spp. | 1 |
| *Staphylococcus succinus* | 1 |
| *Stenotrophomonas maltophilia* | 3 |
| *Streptococcus agalactiae* | 2 |
| *Streptococcus anginosus* | 5 |
| *Streptococcus anginosus group* | 1 |
| *Streptococcus gordonii* | 3 |
| *Streptococcus infantis* | 1 |
| *Streptococcus mitis/oralis* | 6 |
| *Streptococcus parasanguinis* | 7 |
| *Streptococcus salivarius* | 1 |
| *Streptococcus sobrinus* | 2 |
| *Streptococcus* spp. | 1 |
| *Streptococcus vestibularis* | 2 |
| *Streptococcus vestibularis/salivarius* | 1 |
| Unidentified spp. | 18 |
| *Veillonella atypica* | 1 |
| *Veillonella dispar* | 4 |
| *Veillonella parvula* | 5 |

**Table S3** Comparison of clinically-directed culture results of respiratory secretions to Illumina MiSeq 16S rRNA marker-gene sequencing and extended-quantitative culture results.

| Participant ID | VAP diagnosis | Clinically-directed culture of respiratory secretions | Illumina MiSeq 16S rRNA marker-gene sequencing  (RA, %) | Extended-quantitative culture (RA, %) |  |
| --- | --- | --- | --- | --- | --- |
| BCH001 | Yes | *Pseudomonas aeruginosa* | *P. aeruginosa* (0.42) | Not found |  |
| BCH002 | No | *Enterobacter cloacae* | Not found | *E. cloacae* complex (18.37) |  |
| BCH004* | No | No growth | *Staphylococcus aureus* (0.53) | *S. aureus* (6.80) |  |
| BCH005 | No | *S. aureus* | *S. aureus* (95.94) | *S. aureus* (100) |  |
| BCH006 | No | *Escherichia coli* | *Escherichia* (0.05) | Not found |  |
| BCH009 | No | *Haemophilus influenzae*  *S. aureus* | *H. influenzae* (21.07)  *S. aureus* (22.67) | *H. influenzae* (2.74)  *S. aureus* (0.31) |  |
| BCH016 | No | *E. coli* | *Escherichia* (0.14) | *E. coli* (83.33) |  |
| BCH023 | No | *Candida albicans* | Fungi not targeted | ETT not cultured |  |
| BCH025 | No | *Hafnia alvei* | Sequencing threshold not met (<10,000 reads) | *H. alvei* (60.93) |  |
| BCH026 | No | *H. influenzae* | *H. influenzae* (13.09) | Not found |  |
| BCH032 | No | *Stenotrophomonas maltophilia* | *S. maltophilia* (96.85) | ETT not cultured |  |
| BCH034 | No | *Klebsiella pneumoniae* | *Klebsiella* (0.95) | ETT not cultured |  |
| BCH037 | No | *S. aureus* | *S. aureus* (83.57) | *S. aureus* (70.35) |  |
| RVH001* | No | No growth | *H. influenzae* (2.38) | No recognised pathogen |  |
| RVH002 | No | *E. coli*  *C. albicans* | *Escherichia* (98.34)  Fungi not targeted | ETT not cultured |  |
| RVH004 | No | *K. pneumoniae* | *Klebsiella* (1.47) | *K. pneumoniae* (33.62) |  |
| RVH007 | No | *Klebsiella pneumoniae* | *Klebsiella* (47.78) | Not found |  |
| RVH008 | No | *E. coli* | *Escherichia* (2.42) | Not found |  |
| RVH009* | No | No growth | *H. influenzae* (14.42) | *C. albicans* (100) |  |
| RVH011 | Yes | *Proteus mirabilis* | *P. mirabilis* (20.72) | ETT not cultured |  |
| RVH013 | Yes | *H. influenzae* | *H. influenzae* (15.87) | Not found |  |
| RVH015 | Yes | *K. pneumoniae* | *Klebsiella* (2.50) | ETT not cultured |  |
| RVH017 | Yes | *E. cloacae* | Not found | *E. cloacae* complex (100) |  |
| RVH019 | No | *S. aureus* | *S. aureus* (9.58) | Not found |  |
| RVH021 | No | *P. aeruginosa* | *P. aeruginosa* (23.18) | *P. aeruginosa* (100) |  |
| RVH023 | No | *H. influenzae* | *H. influenzae* (1.04) | Not found |  |
| RVH027 | No | *C. albicans* | Fungi not targeted | ETT not cultured |  |
| RVH028 | No | *S. aureus*  *Raoultella ornithinolytica* | *S. aureus* (99.50)  *Raoultella* (0.19) | ETT not cultured |  |
| RVH029 | No | *Enterobacter hormaechei* | Sequencing threshold not met (<10,000 reads) | ETT not cultured |  |

*Definitions:* RA, relative abundance (the higher RA of the two ETT segments analysed by Illumina MiSeq 16S rRNA marker-gene sequencing or extended-quantitative culture is shown).

*Dominant potential pathogen detected by Illumina MiSeq 16S rRNA marker-gene sequencing and/or extended-quantitative culture shown when no growth reported by clinically-directed culture of respiratory secretions.

Final column shading: blue, potential match of Illumina MiSeq 16S rRNA marker-gene sequencing *and* extended-quantitative culture with clinically-directed culture results; orange, potential match of Illumina MiSeq 16S rRNA marker-gene sequencing *or* extended-quantitative culture with clinically-directed culture results; grey, potential match of Illumina MiSeq 16S rRNA marker-gene sequencing with clinically-directed culture results (endotracheal tube not cultured); white, no match of either microbial community profiling result with clinically-directed culture results (various reasons).

**Table S4** Summary of antimicrobial resistance amongst selected endotracheal tube biofilm isolates.

| Antibiotic | No. (%) classed as resistant | | | | | | | | |
| --- | --- | --- | --- | --- | --- | --- | --- | --- | --- |
|  | Enterobacterales*  (n=25) | *Pseudomonas aeruginosa* (n=1) | *Stenotrophomonas maltophilia*  (n=2) | *Staphylococcus aureus*  (n=7) | *Enterococcus*  *faecium*  (n=3) | *Streptococcus agalactiae*  (n=1) | *Streptococcus anginosus* group (n=6) | *Haemophilus influenzae* (n=3) | Anaerobic bacteria^‡^  (n=11) |
| Ampicillin | 20 (80.00) | NA | NA | NA | 2 (66.67) | NA | NA | 2 (66.67) | 11 (100) |
| Aztreonam | 5 (20.00) | 0 (0) | NA | NA | NA | NA | NA | NA | NA |
| Benzylpenicillin | NA | NA | NA | 5 (71.42) | NA | 0 (0) | 0 (0) | NA | 4 (36.36) |
| Ceftriaxone | 3 (12.00) | NA | NA | NA | NA | NA | 0 (0) | 1 (33.33) | NA |
| Cefoxitin | NA | NA | NA | 0 (0)^†^ | NA | NA | NA | NA | NA |
| Cefuroxime | 4 (16.00) | NA | NA | NA | NA | NA | 0 (0) | 0 (0) | NA |
| Ciprofloxacin | 0 (0) | 0 (0) | NA | 0 (0) | NA | NA | NA | 1 (33.33) | NA |
| Clindamycin | NA | NA | NA | 0 (0) | NA | 0 (0) | 0 (0) | NA | 3 (27.27) |
| Co-amoxiclav | 7 (28.00) | NA | NA | NA | NA | NA | NA | 1 (33.33) | NA |
| Co-trimoxazole | 3 (12.00) | NA | 0 (0) | 1 (14.29) | NA | 0 (0) | NA | 0 (0) | NA |
| Erythromycin | NA | NA | NA | 0 (0) | NA | 0 (0) | NA | NA | NA |
| Gentamicin | 0 (0) | NA | NA | 0 (0) | NA | NA | NA | NA | NA |
| Linezolid | NA | NA | NA | 0 (0) | 0 (0) | 0 (0) | NA | NA | NA |
| Meropenem | 2 (8.00) | 0 (0) | NA | NA | NA | NA | NA | 0 (0) | 5 (45.45) |
| Metronidazole | NA | NA | NA | NA | NA | NA | NA | NA | 0 (0) |
| Piperacillin/tazobactam | 19 (76.00) | 1 (100) | NA | NA | NA | NA | NA | NA | 0 (0) |
| Rifampicin | NA | NA | NA | 0 (0) | NA | 0 (0) | NA | 1 (33.33) | NA |
| Teicoplanin | NA | NA | NA | NA | 1 (33.33) | 0 (0) | NA | NA | NA |
| Tetracycline | NA | NA | NA | 0 (0) | NA | 1 (100) | NA | NA | NA |
| Vancomycin | NA | NA | NA | NA | 2 (66.67) | 0 (0) | NA | NA | NA |

**Escherichia coli* (n=6), *Klebsiella oxytoca* (n=3), *Klebsiella pneumoniae* (n=1), *Enterobacter cloacae* complex (n=4), *Hafnia alvei* (n=1), *Pantoea agglomerans* (n=7), *Pantoea septica* (n=1), *Proteus mirabilis* (n=1), *Serratia liquefaciens* (n=1).

^†^Indicates meticillin-sensitive *Staphylococcus aureus.*

^‡^*Prevotella melaninogenica* (n=2), *Prevotella denticola* (n=1), *Prevotella histicola* (n=5), *Prevotella nanceiensis* (n=1), *Prevotella salivae* (n=1), *Prevotella nigrescens* (n=1).

**References**

1. Carson L, Gorman SP, Gilmore BF. The use of lytic bacteriophages in the prevention and eradication of biofilms of *Proteus mirabilis* and *Escherichia coli*. FEMS Immunol Med Microbiol. 2010;59(3):447-55.

2. Whiley RA, Fleming EV, Makhija R, Waite RD. Environment and colonisation sequence are key parameters driving cooperation and competition between *Pseudomonas aeruginosa* cystic fibrosis strains and oral commensal streptococci. PLoS One. 2015;10(2):e0115513.

3. Earth Microbiome Project. 16S Illumina Amplicon Protocol. 2025. <https://earthmicrobiome.org/protocols-and-standards/16s/>

4. Davis NM, Proctor DM, Holmes SP, Relman DA, Callahan BJ. Simple statistical identification and removal of contaminant sequences in marker-gene and metagenomics data. Microbiome. 2018;6(1):226.

5. Lane D. Nucleic acid techniques in bacterial systematics. E Stackebrandt MG, editor. New York: Wiley; 1991.

6. Edwards U, Rogall T, Blocker H, Emde M, Bottger EC. Isolation and direct complete nucleotide determination of entire genes. Characterization of a gene coding for 16S ribosomal RNA. Nucleic Acids Res. 1989;17(19):7843-53.

7. Muyzer G, Teske A, Wirsen CO, Jannasch HW. Phylogenetic relationships of *Thiomicrospira* species and their identification in deep-sea hydrothermal vent samples by denaturing gradient gel electrophoresis of 16S rDNA fragments. Arch Microbiol. 1995;164(3):165-72.

8. Tarumoto N, Sakai J, Kodana M, Kawamura T, Ohno H, Maesaki S. Identification of disseminated Cryptococcosis using MALDI-TOF MS and clinical evaluation. Med Mycol J. 2016;57(3):E41-6.

9. Rousseeuw PJ. Silhouettes: A graphical aid to the interpretation and validation of cluster analysis. Journal of Computational and Applied Mathematics. 1987;20:53-65.
